# Supplementary material for: Thermodynamic and Kinetic Sequence Selection in Enzyme-Free Polymer Self-Assembly inside a Non-equilibrium RNA Reactor
Source: Life (Basel). 2022 Apr 10;12(4):567. doi: 10.3390/life12040567 (PMC9032526; doi:10.3390/life12040567)
Supplement: Supplementary file 1 [file life-12-00567-s001.zip › separate_supplement.pdf]

Thermodynamic and Kinetic Sequence Selection in Enzyme-Free Polymer Self-Assembly Inside a Non-Equilibrium RNA Reactor

— Supplemental Material —

Tobias Göppel[1], Joachim H. Rosenberger[1], Berhard Altaner[1] and Ulrich Gerland[1]

[1]Physics of Complex Biosystems, Technical University of Munich, 85748 Garching, Germany

S1. REFERENCE MODEL WITHOUT ENERGETIC BIAS AND KINETIC STALLING — DETAILS

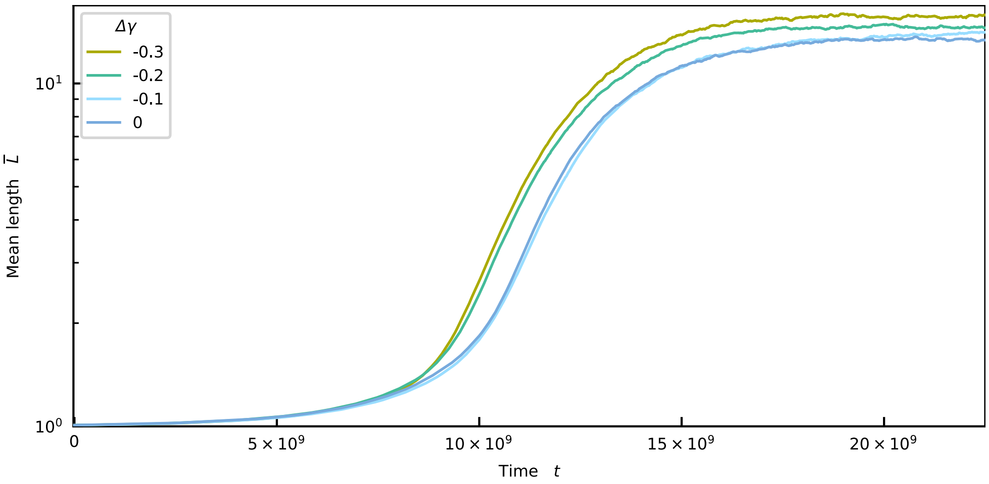

FIG. S1. Log–linear plot of the evolution of the mean length $\overline{L}$ for $\sigma_1 = \sigma_2 = 1$ and various values of $\Delta\gamma$ (see also Figure 3 in the main text). After a short lag phase, the initial growth of $\overline{L}$ is exponential.

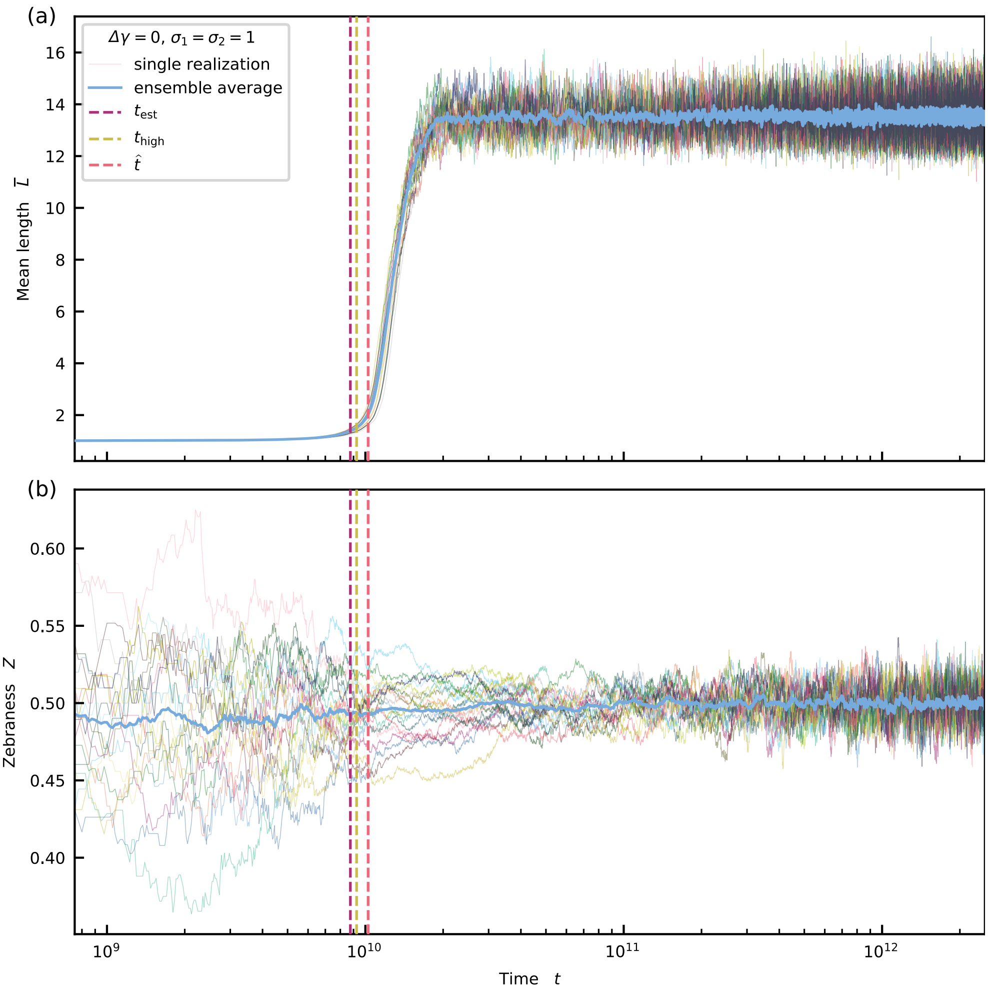

FIG. S2. (a) and (b) Evolution of the mean length $\overline{L}$ and system-level zebraness Z for $\Delta\gamma = 0$ and $\sigma_1 = \sigma_2 = 1$ (see also Figure 3 in the main text). The relative deviations of t_{est} from t_{high} and $\widehat{t}$ are 5.4% and 14.6%.

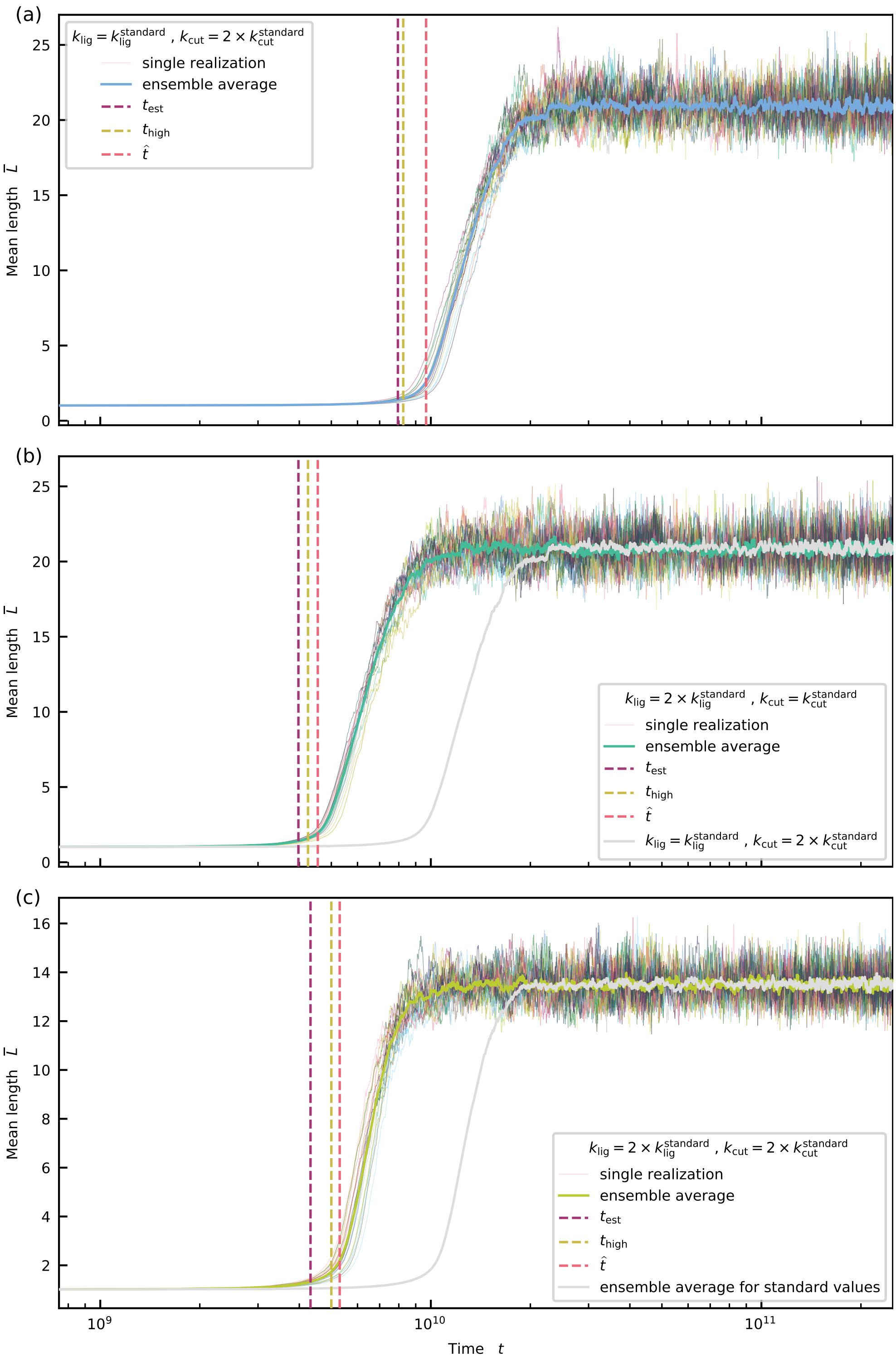

FIG. S3. Evolution of the mean length $\overline{L}$ for $\Delta\gamma = 0$ and $\sigma_1 = \sigma_2 = 1$ for k_{lig} and k_{cut} different from the standard values $k_{\text{lig}}^{\text{standard}}$ and $k_{\text{cut}}^{\text{standard}}$ given in Table 2 (see also Figure 3 in the main text). (a) Reducing the rate of hydrolysis by a factor of two moves the onset slightly to the left. The relative deviations of t_{est} from t_{high} and $\widehat{t}$ are 3.6% and 17.8%. (b) Multiplying the ligation rate by a factor of two and leaving the hydrolysis rate unchanged shifts the onset of growth to the left $\widehat{t}$. The relative deviations of t_{est} from t_{high} and $\widehat{t}$ are 6.4% and 12.6%. (c) Doubling both the ligation and hydrolysis rate shifts the onset to the left and shortens the time window of the rapid growth phase. The relative deviations of t_{est} from t_{high} and $\widehat{t}$ are 13.5% and 18.4%. (a), (b) and (x) The mean length $\overline{L}$ in the steady-state is the same in the first and the second scenario. In the third scenario, the stationary value of $\overline{L}$ is the same as in the standard scenario (grey line). This observation suggests that $\overline{L}$ only depends on the ratio of k_{lig} and k_{cut}, i.e., $\overline{L} = \overline{L}\left(k_{\text{lig}}/k_{\text{cut}}\right)$ (see also Figure S16).

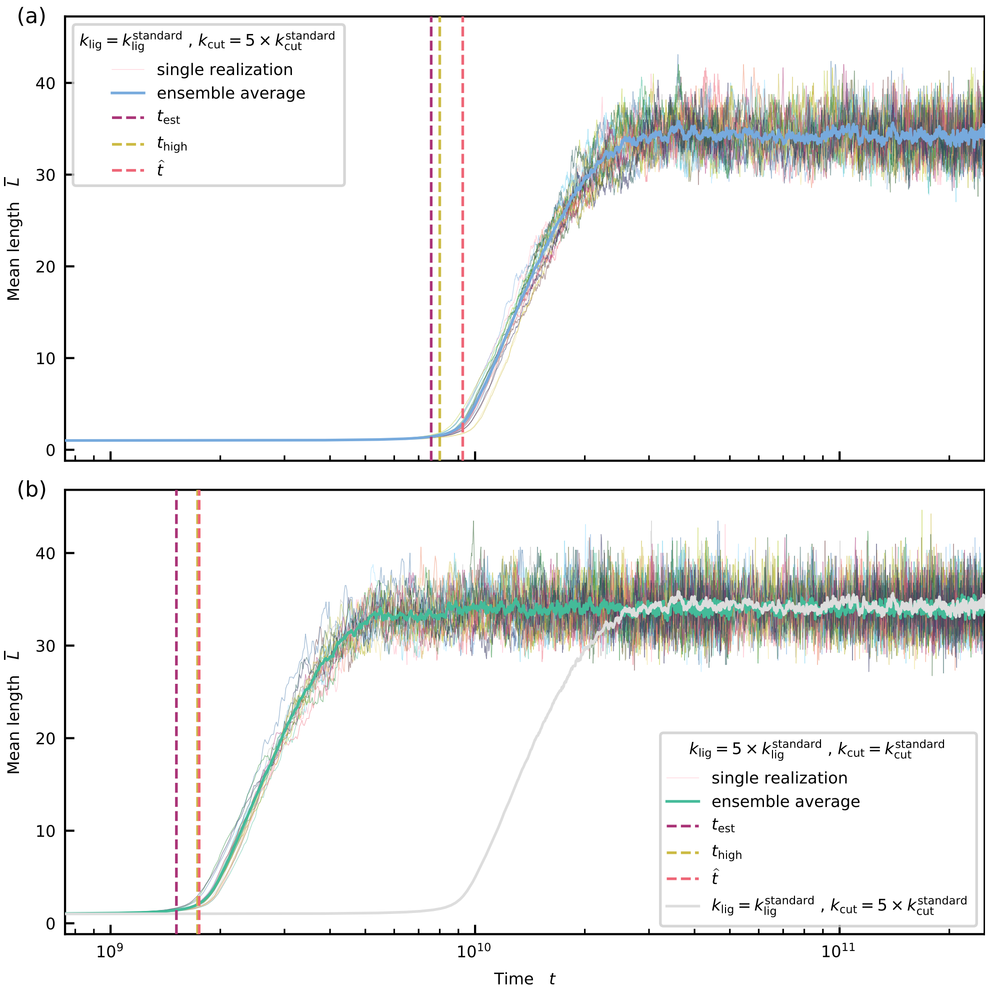

FIG. S4. Evolution of the mean length $\overline{L}$ for $\Delta\gamma = 0$ and $\sigma_1 = \sigma_2 = 1$ for k_{lig} and k_{cut} different from the standard values $k_{\text{lig}}^{\text{standard}}$ and $k_{\text{cut}}^{\text{standard}}$ given in Table 2 (see also Figure 3 in the main text). (a) Reducing the rate of hydrolysis by a factor of five moves the onset slightly to the left. The relative deviations of t_{est} from t_{high} and $\widehat{t}$ are 5.2% and 18.0%. The relative deviations of t_{est} from t_{high} and $\widehat{t}$ are both 13.4%. (b) Multiplying the ligation rate by a factor of five and leaving the hydrolysis rate unchanged shifts the onset to the left $\widehat{t}$. The mean length $\overline{L}$ in the steady-state is the same in the first and the second scenario (see gray line). This observation again suggests that $\overline{L}$ only depends on the ratio of k_{lig} and k_{cut}, i.e., $\overline{L} = \overline{L}\left(k_{\text{lig}}/k_{\text{cut}}\right)$ (see also Figure S16).

S2. ENERGETIC BIAS IN THE ABSENCE OF KINETIC STALLING — DETAILS

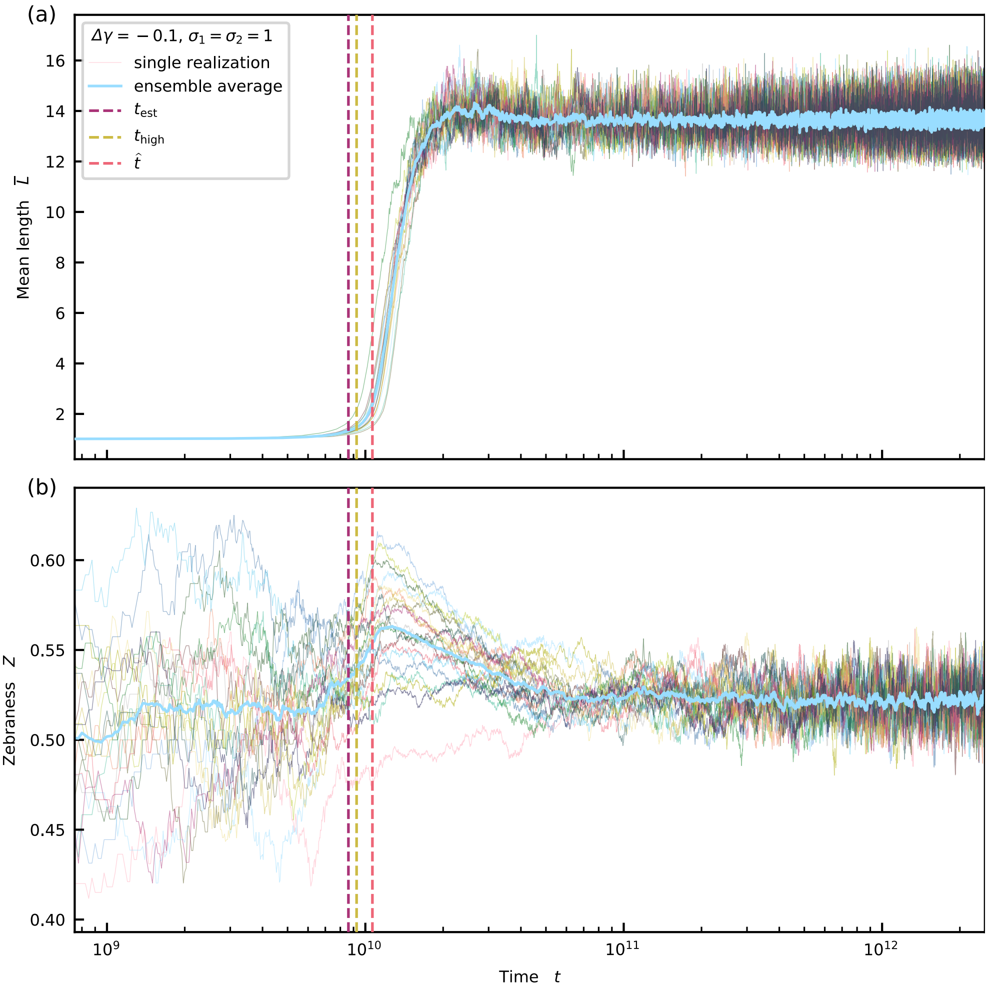

FIG. S5. (a) and (b) Evolution of the mean length $\overline{L}$ and system-level zebraness Z for $\Delta\gamma = -0.1$ and $\sigma_1 = \sigma_2 = 1$ (see also Figure 3 in the main text). The relative deviations of t_{est} from t_{high} and $\widehat{t}$ are 7.0% and 19.2%.

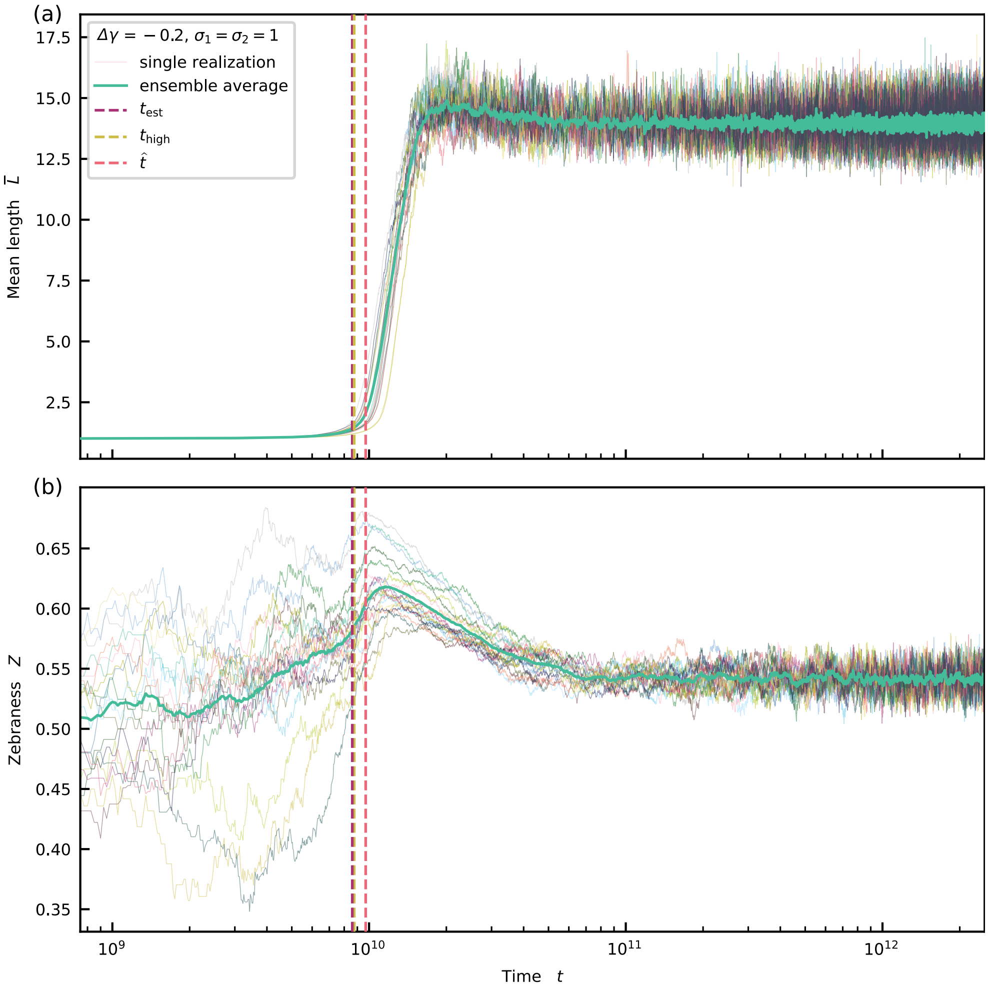

FIG. S6. (a) and (b) Evolution of the mean length $\overline{L}$ and system-level zebraness Z for $\Delta\gamma = -0.2$ and $\sigma_1 = \sigma_2 = 1$ (see also Figure 3 in the main text). The relative deviations of t_{est} from t_{high} and $\widehat{t}$ are 1.7% and 11.3%.

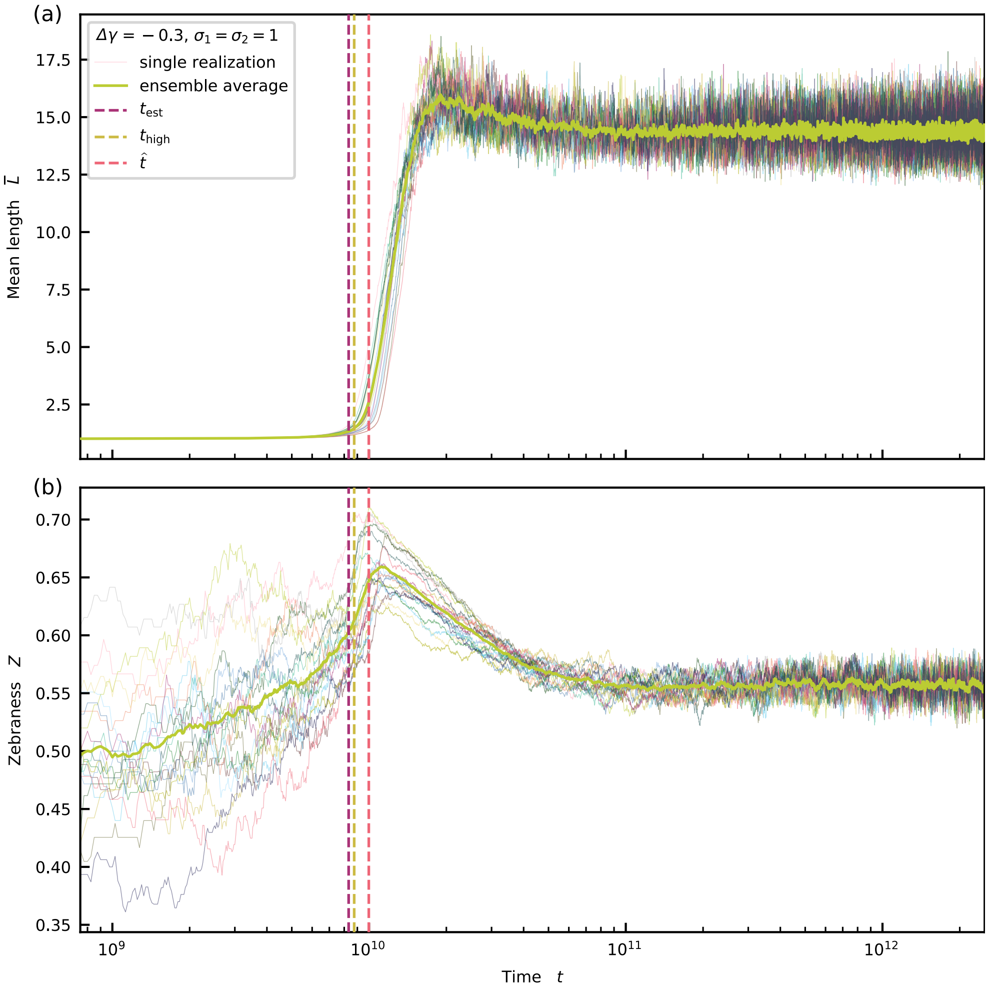

FIG. S7. (a) and (b) Evolution of the mean length $\overline{L}$ and system-level zebraness Z for $\Delta\gamma = -0.3$ and $\sigma_1 = \sigma_2 = 1$ (see also Figure 3 in the main text). The relative deviations of t_{est} from t_{high} and $\widehat{t}$ are 4.9% and 16.5%.

S3. KINETIC STALLING IN THE ABSENCE OF ENERGETIC BIAS — DETAILS

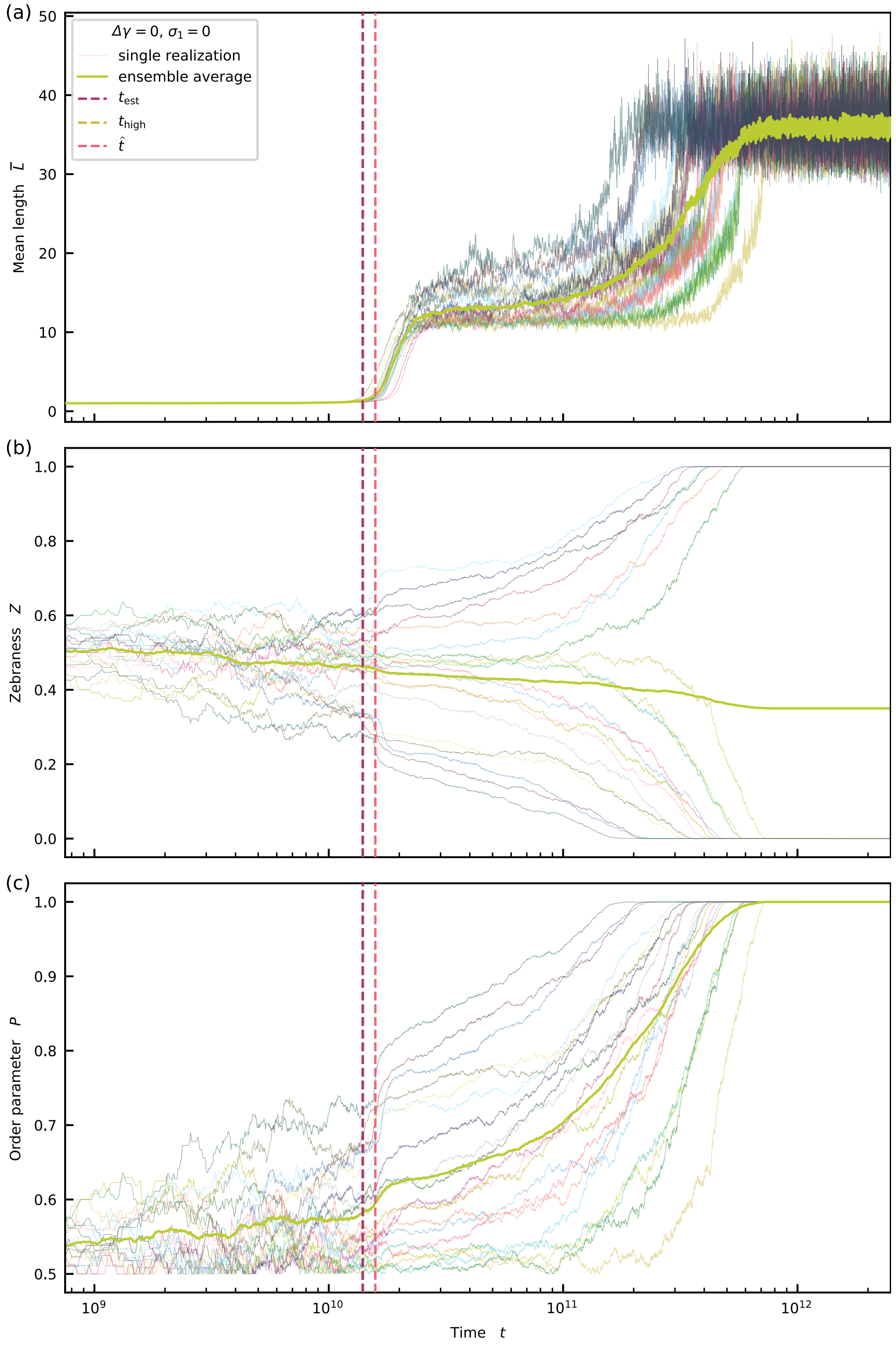

FIG. S8. (a), (b) and (c) Evolution of the mean length $\overline{L}$, system-level zebraness Z, and sequence order parameter P for $\Delta\gamma = 0$ and $\sigma_1 = 0$ (strong kinetic stalling). All trajectories reach a pure states ($Z = 0, 1$). The relative deviations of t_{est} from t_{high} and $\widehat{t}$ are 0.3% and 11.7%. (See also Figure 4 in the main text.)

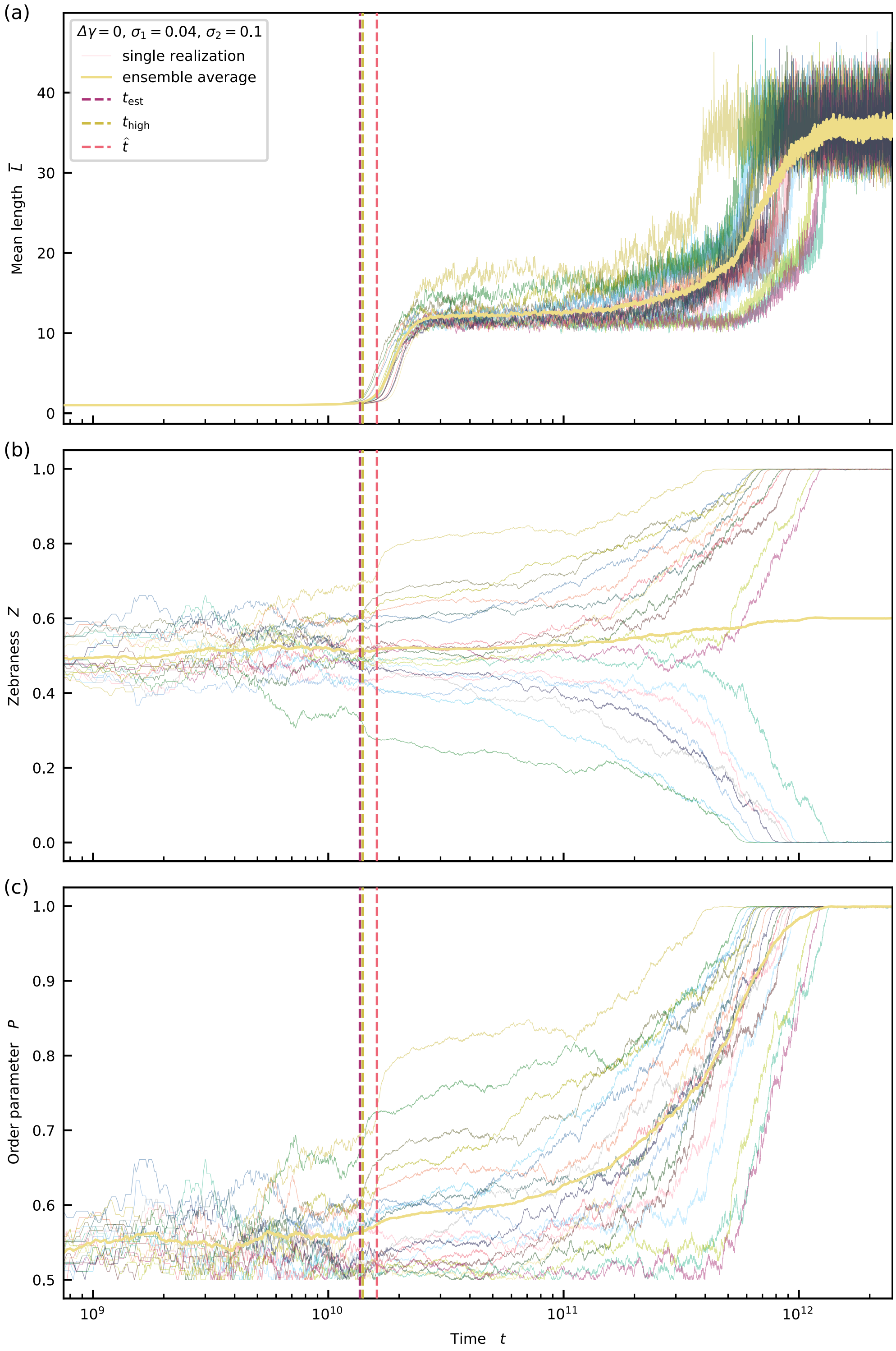

FIG. S9. (a), (b) and (c) Evolution of the mean length $\overline{L}$ and system-level zebraness Z, and sequence order parameter P for $\Delta\gamma = 0$ and $\sigma_1 = 0.04$ and $\sigma_1 = 0.1$ (strong kinetic stalling). All trajectories reach a pure states ($Z = 0, 1$). The relative deviations of t_{est} from t_{high} and $\widehat{t}$ are 2.6% and 15.4%. (See also Figure 4 in the main text.)

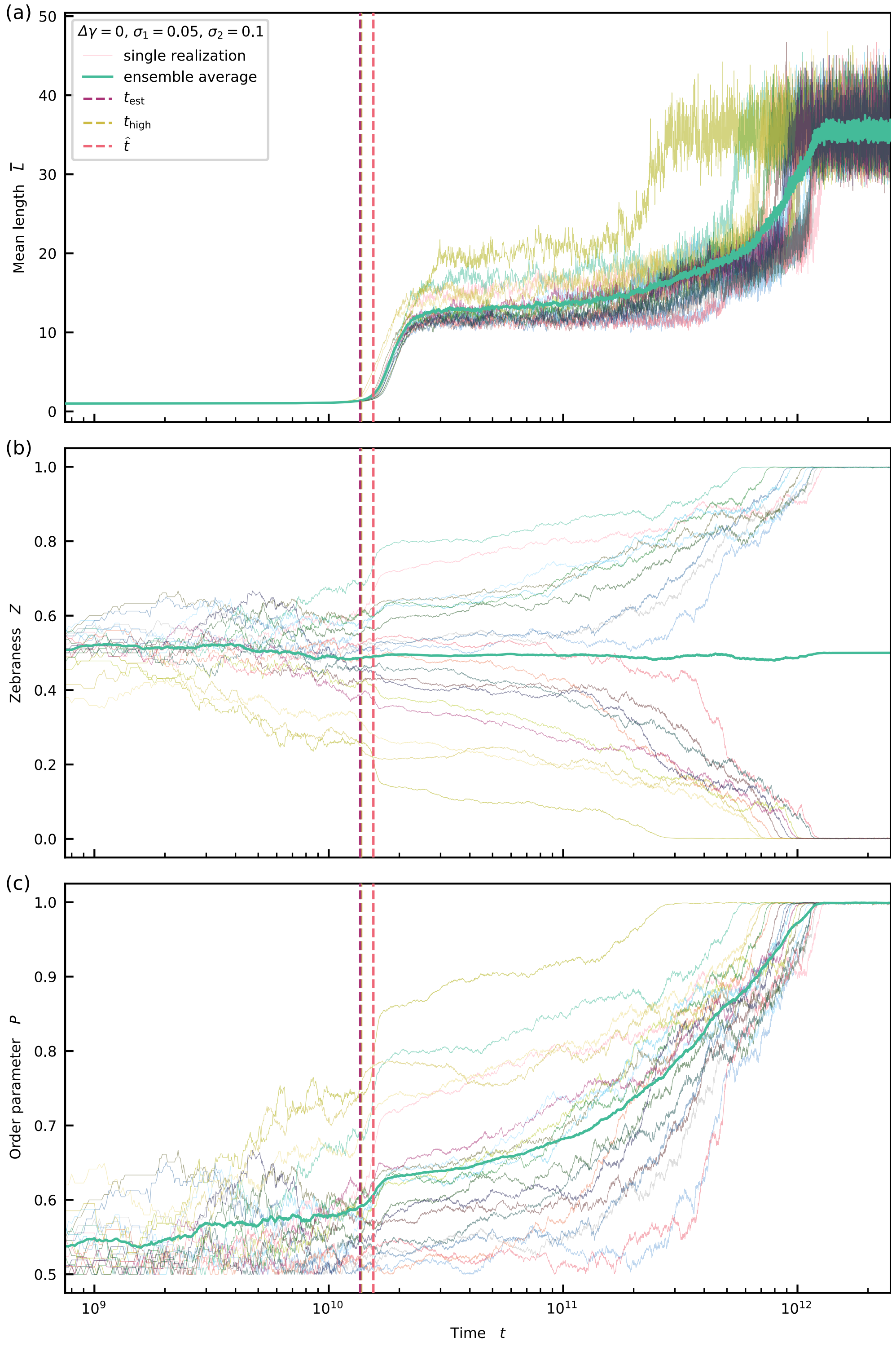

FIG. S10. (a), (b) and (c) Evolution of the mean length $\overline{L}$ and system-level zebraness Z, and sequence order parameter P for $\Delta\gamma = 0$ and $\sigma_1 = 0.05$ and $\sigma_1 = 0.1$ (strong kinetic stalling). All trajectories reach a pure states ($Z = 0, 1$). The relative deviations of t_{est} from t_{high} and $\widehat{t}$ are 1.6% and 12.7%. (See also Figure 4 in the main text.)

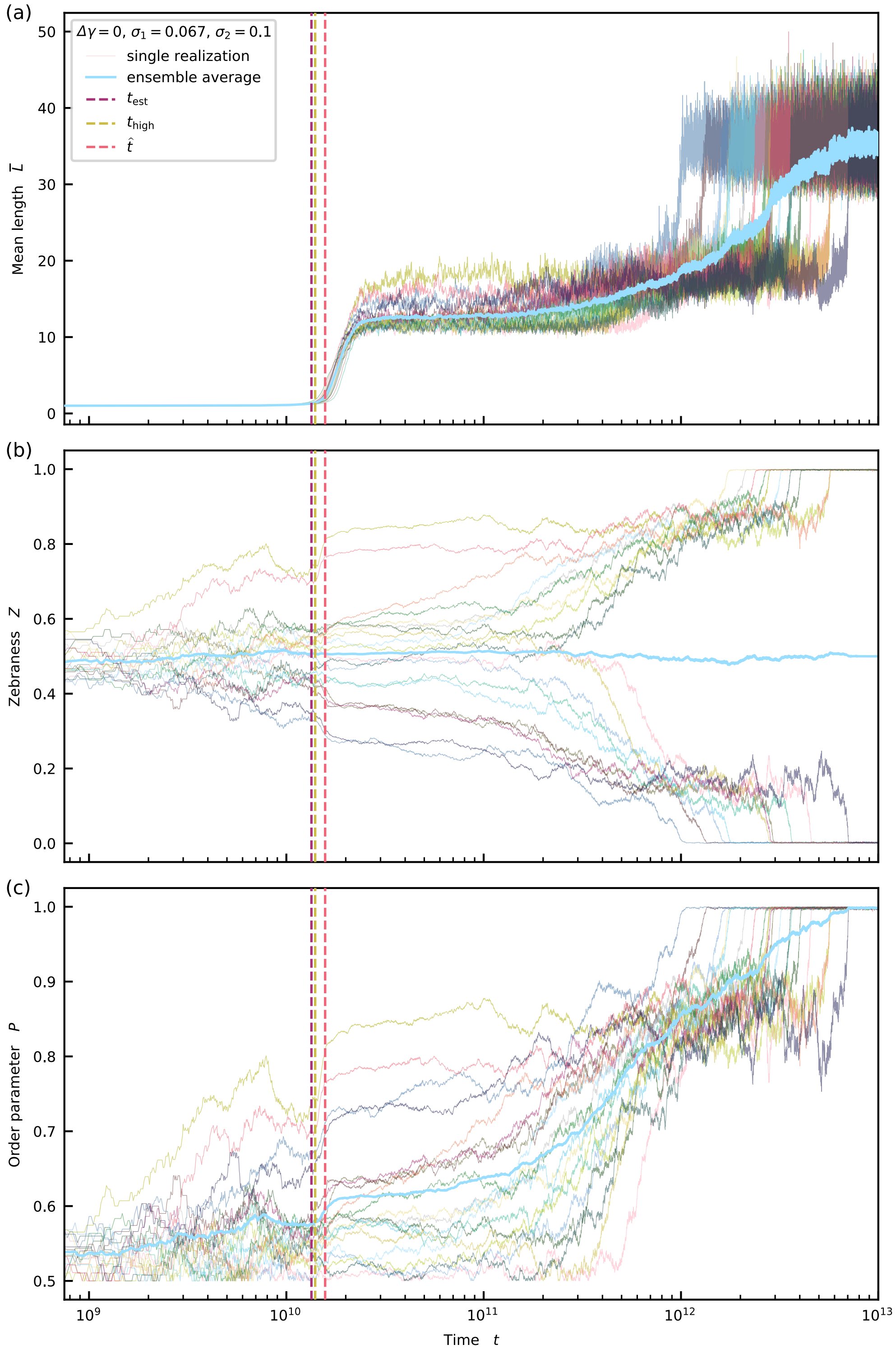

FIG. S11. (a), (b) and (c) Evolution of the mean length $\overline{L}$ and system-level zebraness Z, and sequence order parameter P for $\Delta\gamma = 0$ and $\sigma_1 = 0.067$ and $\sigma_1 = 0.1$ (strong kinetic stalling). All trajectories reach a pure states ($Z = 0, 1$). Note that the system needs approximately ten times longer to reach the steady-state for $\sigma_1 = 0.067$ compared to $\sigma_1 = 0, 0.04, 0.05$ (see also Figure 4 in the main text and Figures S8–S10. The relative deviations of t_{est} from t_{high} and $\widehat{t}$ are 4.2% and 14.8%.

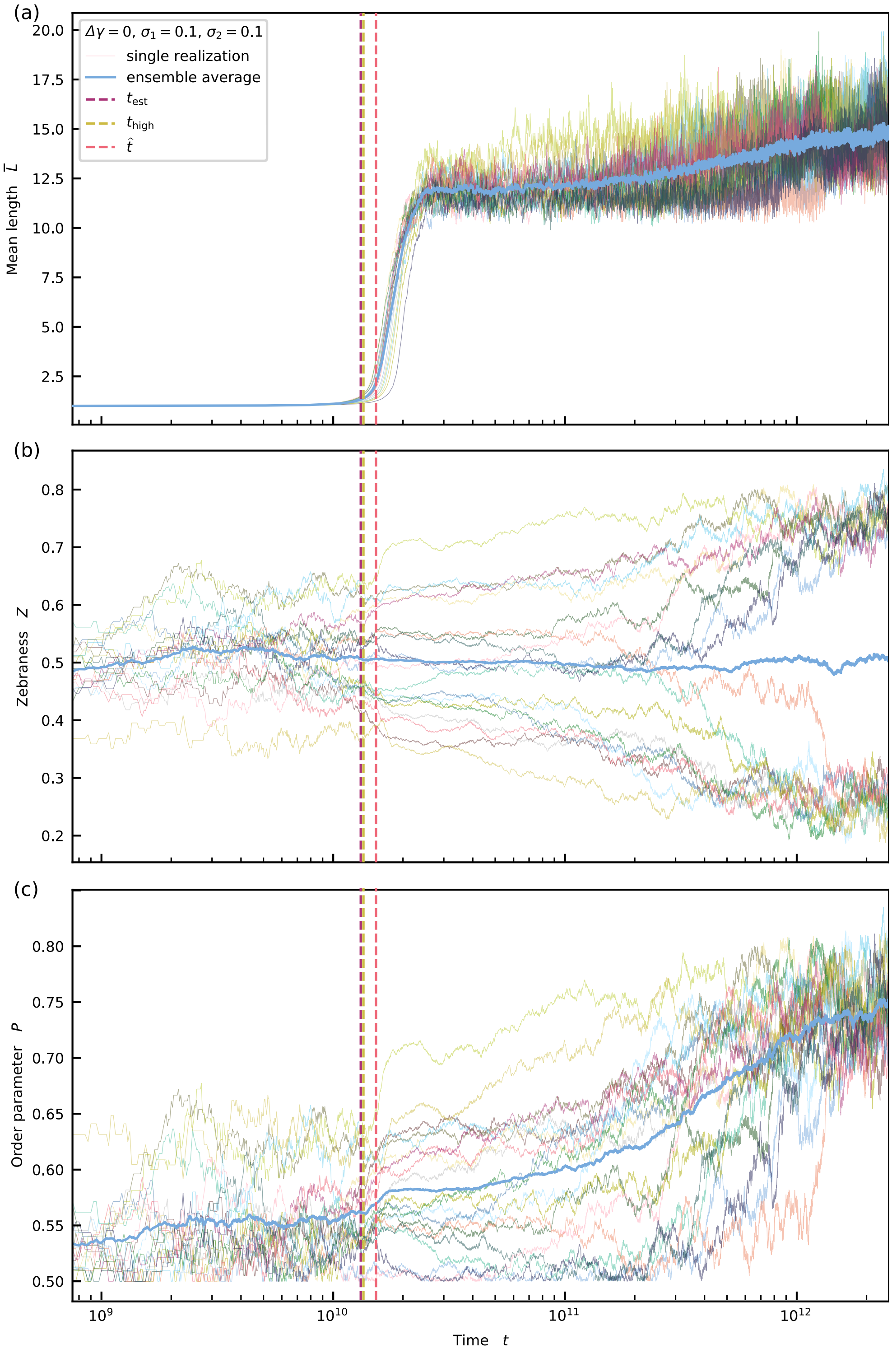

FIG. S12. (a), (b) and (c) Evolution of the mean length $\overline{L}$ and system-level zebraness Z, and sequence order parameter P for $\Delta\gamma = 0$ and $\sigma_1 = 0.1$ and $\sigma_1 = 0.1$ (weak kinetic stalling). In the weak kinetic stalling scenario, the trajectories do not reach a pure states ($Z = 0, 1$) for $t \to \infty$. The relative deviations of t_{est} from t_{high} and $\widehat{t}$ are 2.6% and 14.0%. (See also Figure 4 in the main text).

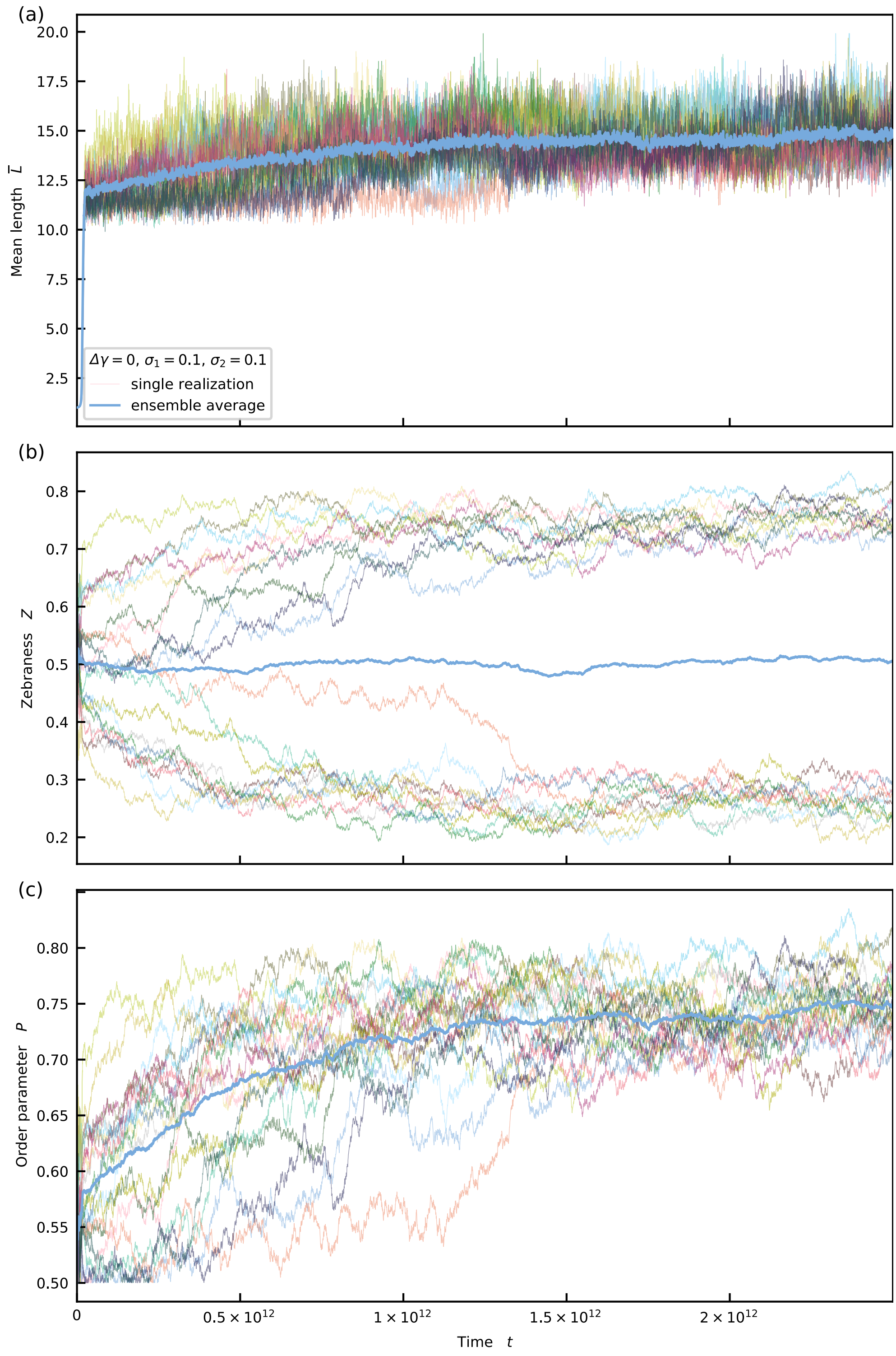

FIG. S13. (a), (b) and (c) Visualization of the evolution of the mean length $\overline{L}$ and system-level zebraness Z, and sequence order parameter P for $\Delta\gamma = 0$ and $\sigma_1 = 0.1$ and $\sigma_1 = 0.1$ (weak kinetic stalling) with a linear x-axis. (See also Figure 4 in the main text an Figure S12).

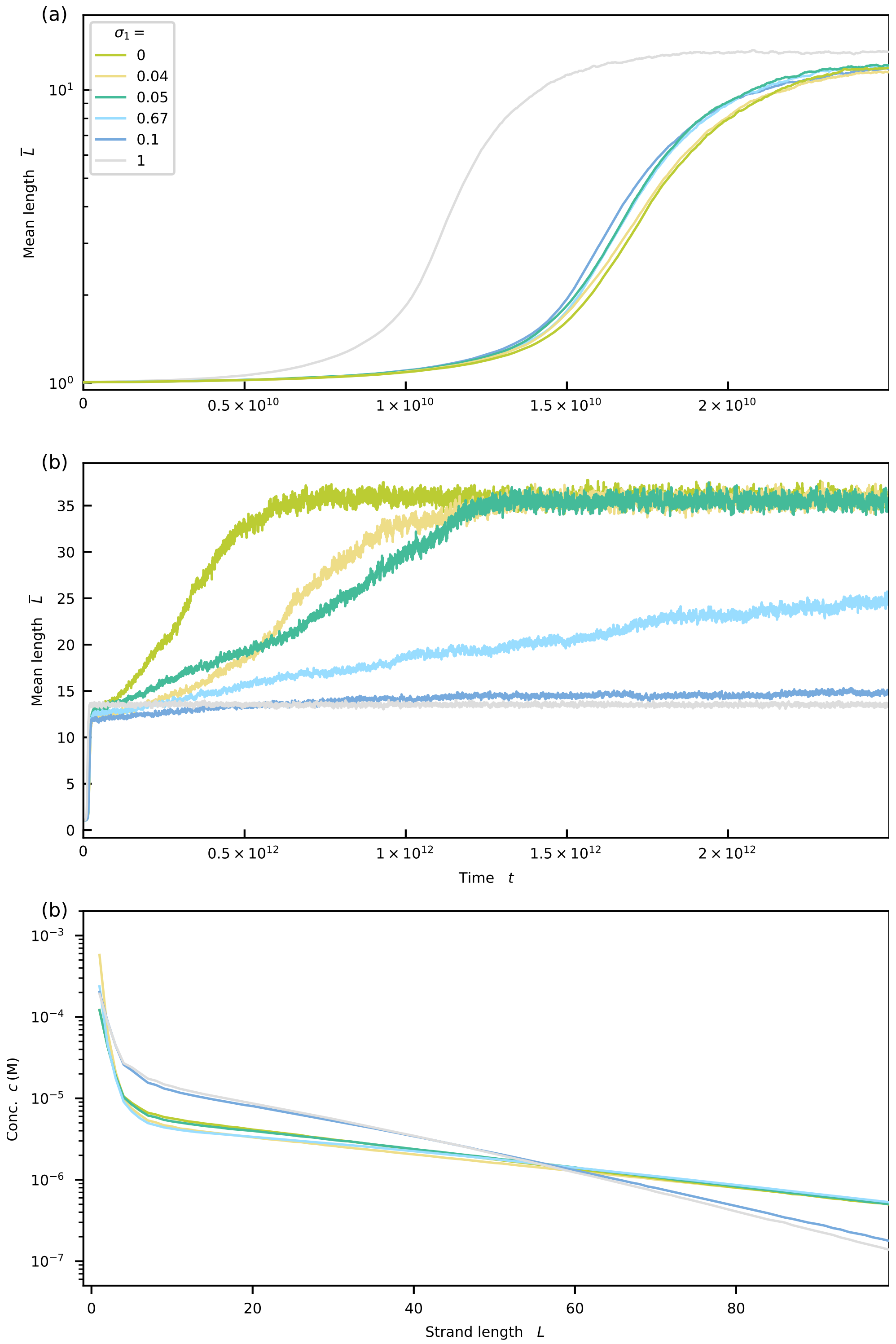

FIG. S14. (a) and (b) The log–linear and linear–linear plots of the evolution of the mean length $\overline{L}$ for $\Delta\gamma = 0$ and various values of σ_1 and $\sigma_2 = 0.1$ reveal a approximately exponential first growth phase and a approximately linear second growth phase of $\overline{L}$ (see also Figure 4 in the main text). (c) The steady-state length distribution displays a double-exponential shape.

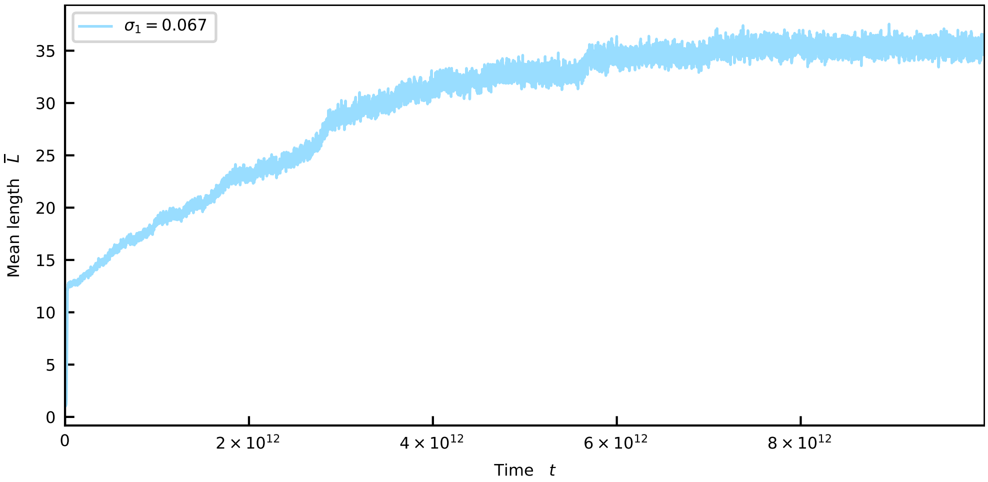

FIG. S15. Linear–linear plot of the evolution of the mean length $\overline{L}$ for $\Delta\gamma = 0$ and $\sigma_1 = 0.067$ and $\sigma_2 = 0.1$ (see also Figure S14 and Figure 4 in the main text)

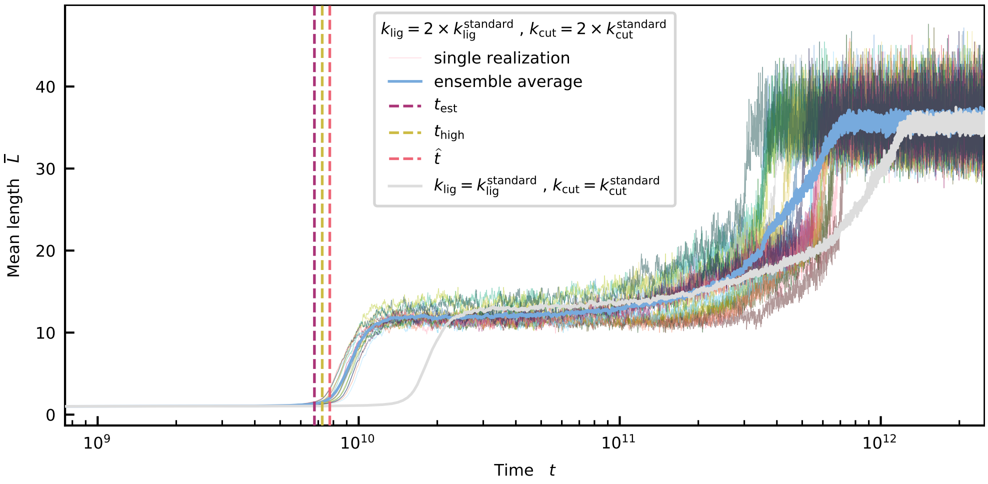

FIG. S16. Evolution of the mean length $\overline{L}$ for $\Delta\gamma = 0.05$ and $\sigma_1 = \sigma_2 = 0.1$ for k_{lig} and k_{cut} twice as large as the standard values $k_{\text{lig}}^{\text{standard}}$ and $k_{\text{cut}}^{\text{standard}}$ given in Table 2 (see also Figure 4 in the main text). Doubling both rates shifts the onset to the left compared to the standard scenario (gray line). The relative deviations of t_{est} from t_{high} and $\widehat{t}$ are 6.7% and 12.7%. The mean length $\overline{L}$ in the stationary-state is the same in the standard scenario. This observation again suggests that $\overline{L}$ only depends on the ratio of k_{lig} and k_{cut}, i.e., $\overline{L} = \overline{L}\left(k_{\text{lig}}/k_{\text{cut}}\right)$

S4. HYDROLYSIS AND STALLING BOOST SEQUENCE SELECTION — DETAILS

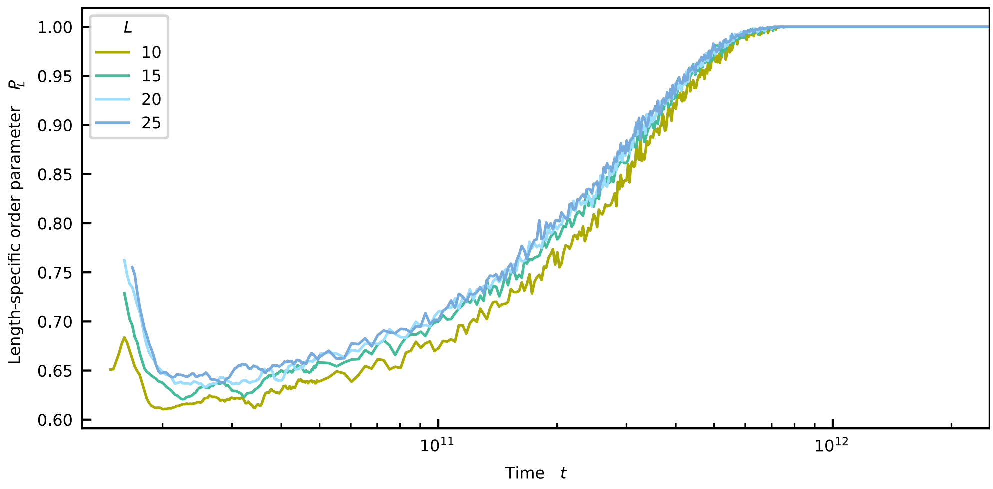

FIG. S17. Evolution of the sequence order parameter P_L of sequences of specific lengths $L = 10, 15, 20, 25$ for $\Delta\gamma = 0$ and $\sigma_1 = 0$. Initially, low molecule numbers lead to an increased sequence order parameter. As more strands of the given lengths form during the first rapid growth phase, their sequences become more random on average, and the P_L decrease. During the second growth phase (see Figure 4 in the main text), existing strands of the given lengths break and new ones are assembled from shorter fragments. The newly assembled strands reflect the average sequence bias of the whole pool. This process becomes self-amplifying. Step by step, all strands with sequences not in line with the average sequence bias get replaced. Eventually, all strands are either fully homogeneous or zebra-like.

S5. ENERGETIC BIAS IN THE PRESENCE OF STRONG KINETIC STALLING — DETAILS

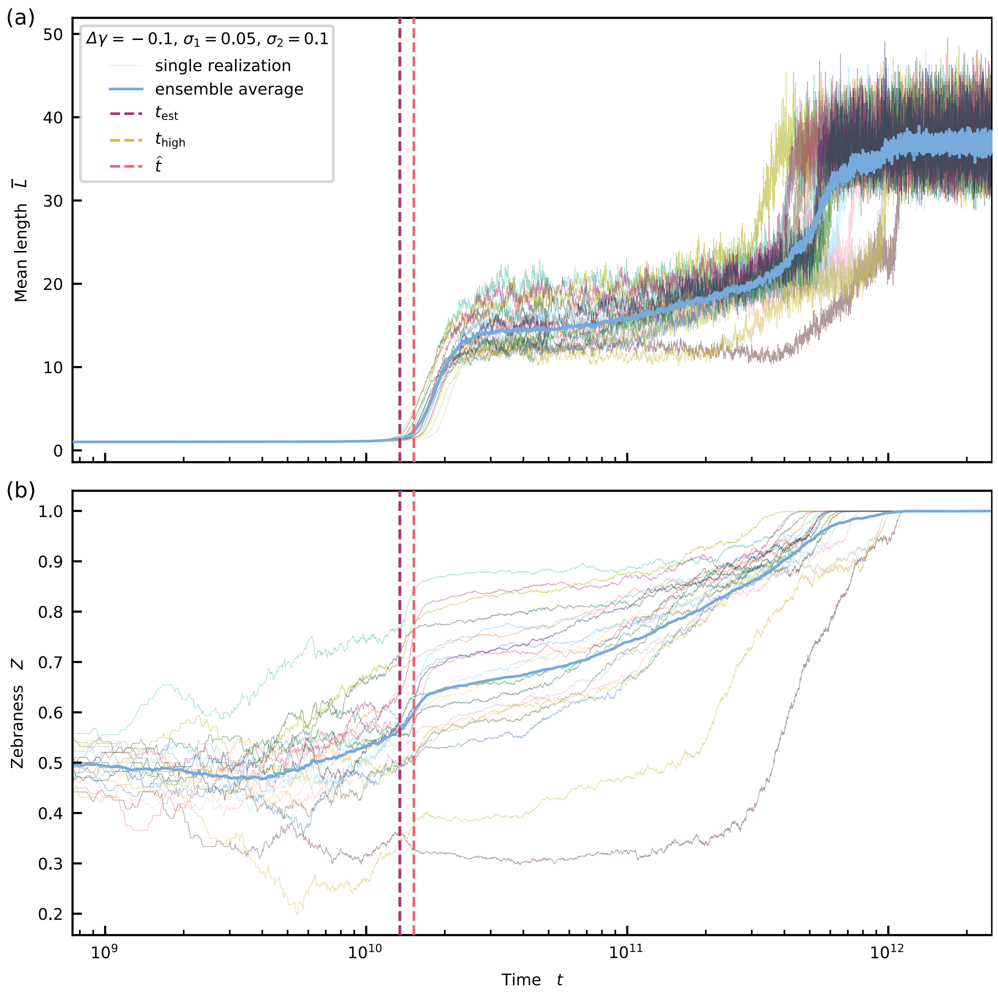

FIG. S18. (a) and (b) Evolution of the mean length $\overline{L}$ and system-level zebraness Z for $\Delta\gamma = -0.1$ and $\sigma_1 = 0.05$ and $\sigma_1 = 0.1$ (strong kinetic stalling). All trajectories reach a pure zebra states ($Z = 1$). The relative deviations of $t_{\rm est}$ from $t_{\rm high}$ and $\widehat{t}$ are 0.4% and 11.9%. (See also Figure 5 in the main text.)

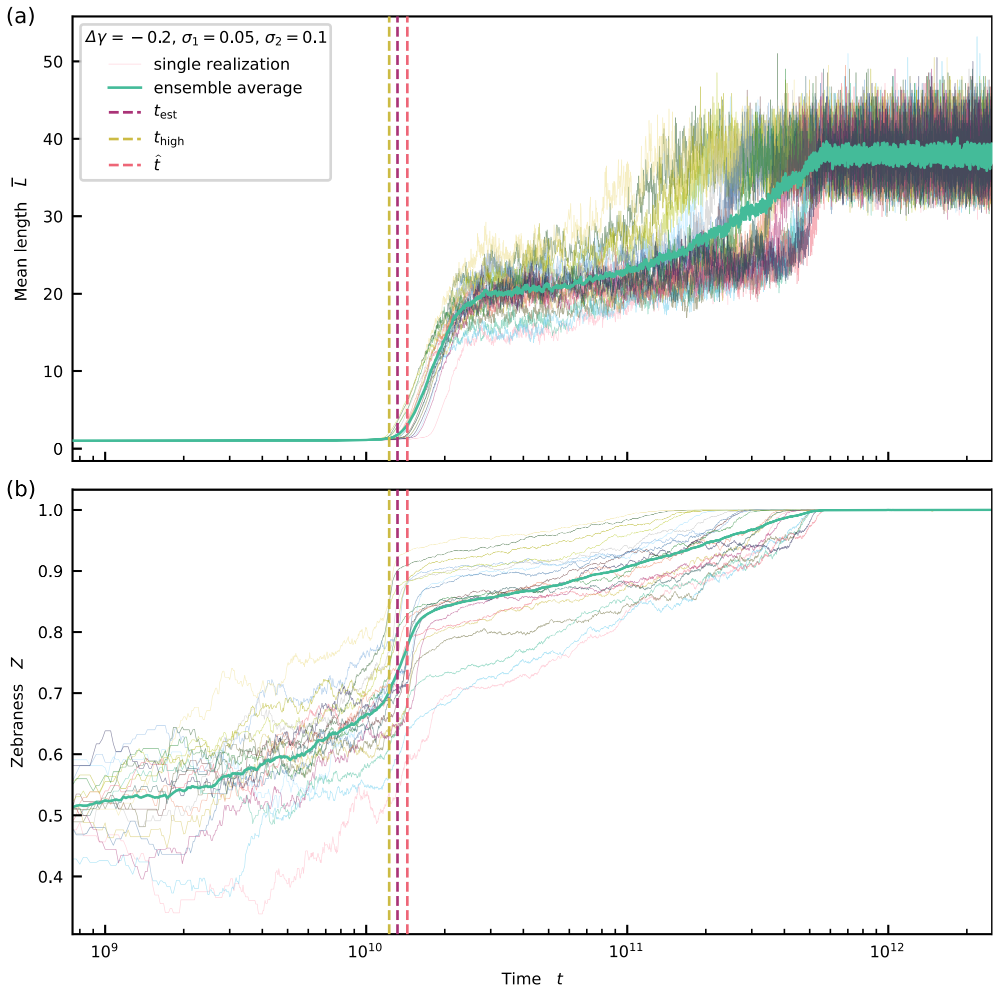

FIG. S19. Evolution of the mean length $\overline{L}$ and system-level zebraness Z for $\Delta\gamma = -0.2$ and $\sigma_1 = 0.05$ and $\sigma_1 = 0.1$ (strong kinetic stalling). All trajectories reach a pure zebra states ($Z = 1$). The relative deviations of t_{est} from t_{high} and $\widehat{t}$ are -7.6% and 8.3%. (See also Figure 5 in the main text.)

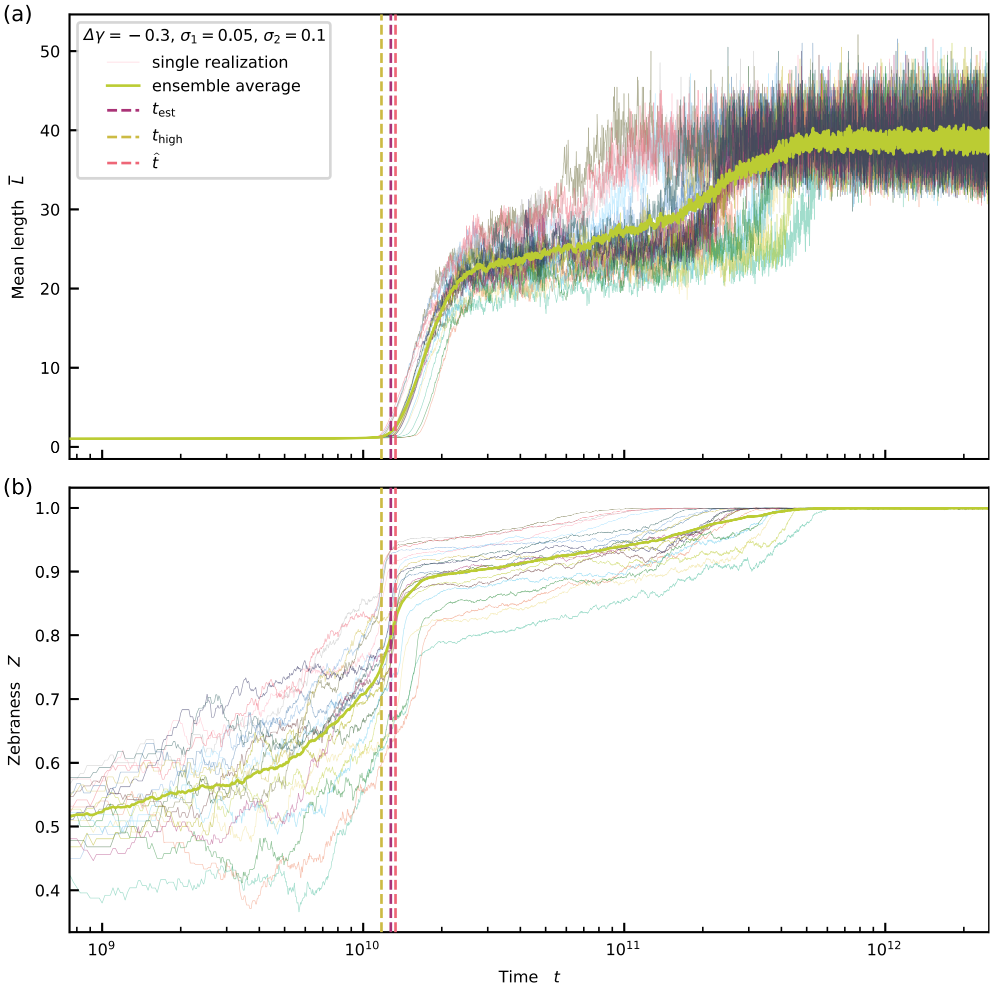

FIG. S20. Evolution of the mean length $\overline{L}$ and system-level zebraness Z for $\Delta\gamma = -0.3$ and $\sigma_1 = 0.05$ and $\sigma_1 = 0.1$ (strong kinetic stalling). All trajectories reach a pure zebra states ($Z = 1$). The relative deviations of t_{est} from t_{high} and $\widehat{t}$ are -8.6% and 4.1%. (See also Figure 5 in the main text.)

S6. ENERGETIC BIAS IN THE PRESENCE OF WEAK KINETIC STALLING — DETAILS

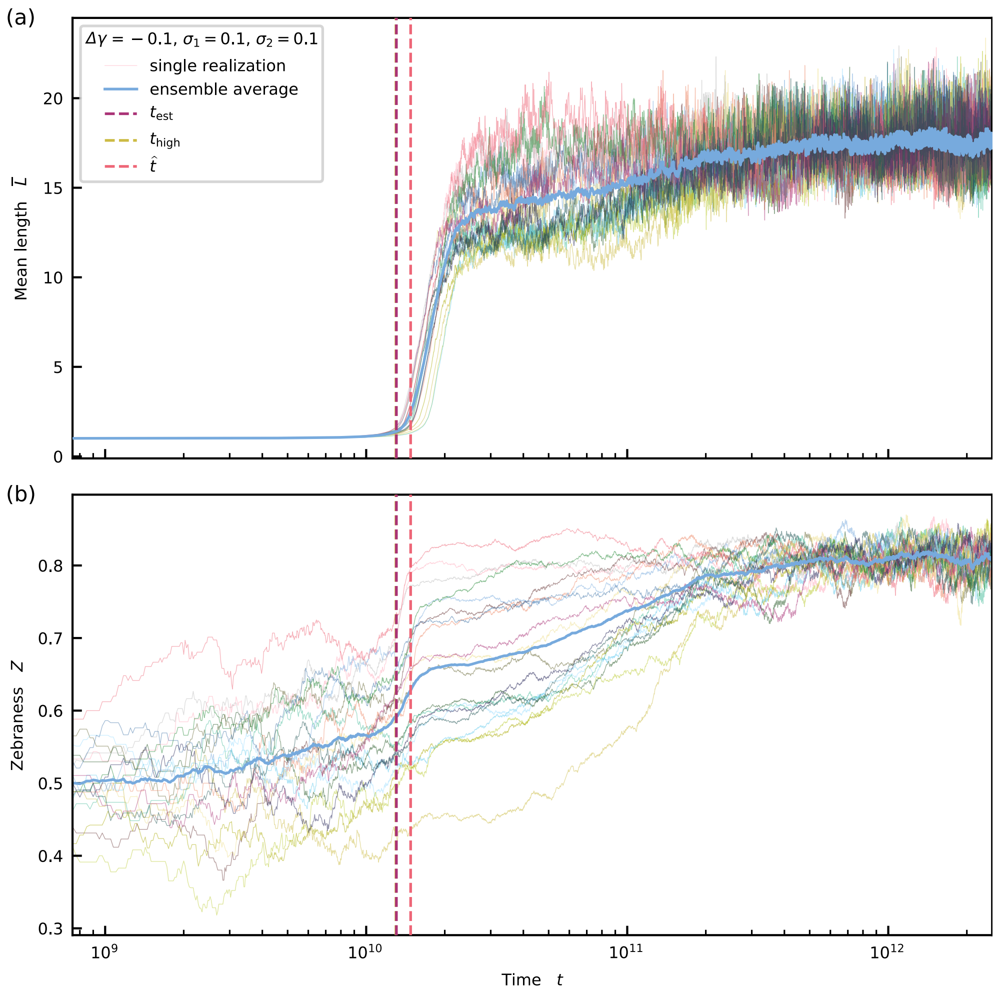

FIG. S21. Evolution of the mean length $\overline{L}$ and system-level zebraness Z for $\Delta\gamma = -0.1$ and $\sigma_1 = \sigma_1 = 0.1$ (weak kinetic stalling). All trajectories reach a non-pure zebra states ($Z < 1$). The relative deviations of $t_{\rm est}$ from $t_{\rm high}$ and $\widehat{t}$ are -0.4% and 11.8%. (See also Figure 5 in the main text.)

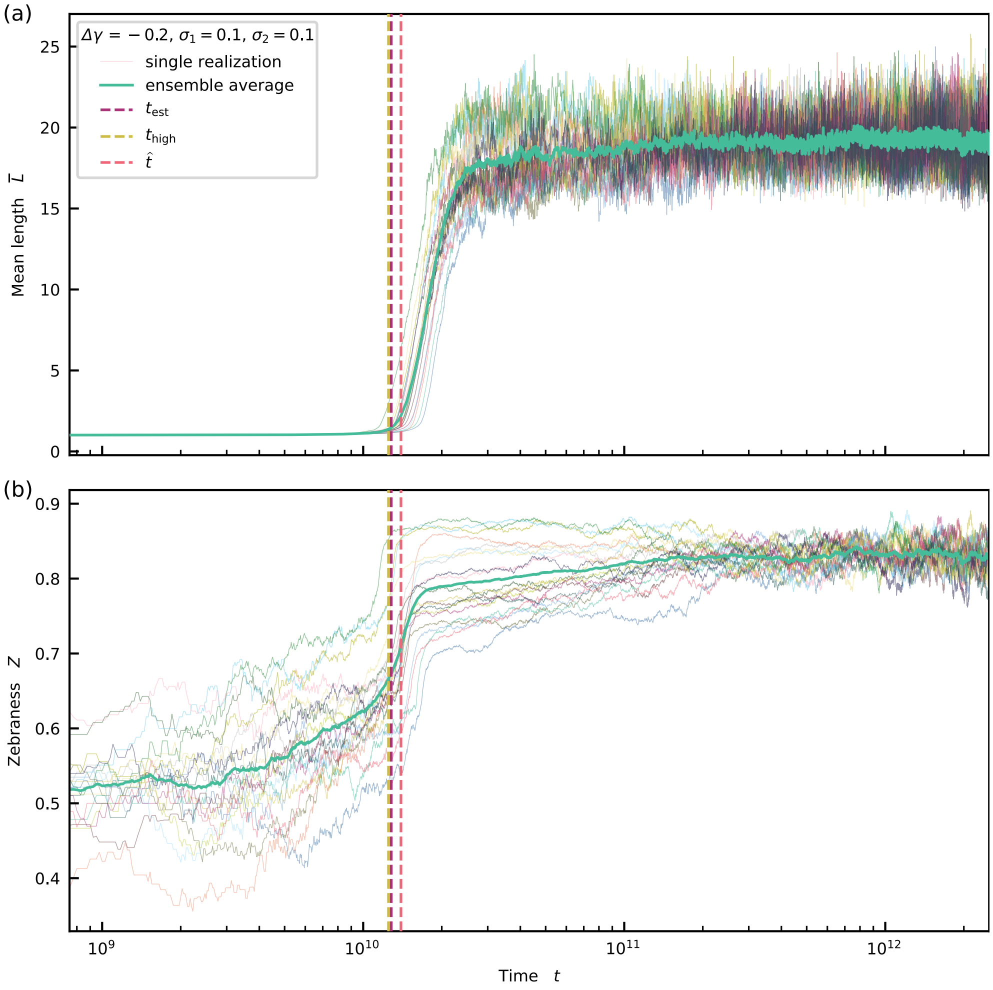

FIG. S22. Evolution of the mean length $\overline{L}$ and system-level zebraness Z for $\Delta\gamma = -0.2$ and $\sigma_1 = \sigma_1 = 0.1$ (weak kinetic stalling). All trajectories reach a non-pure zebra states ($Z < 1$). The relative deviations of t_{est} from t_{high} and $\widehat{t}$ are -2.4% and 8.2%. (See also Figure 5 in the main text.)

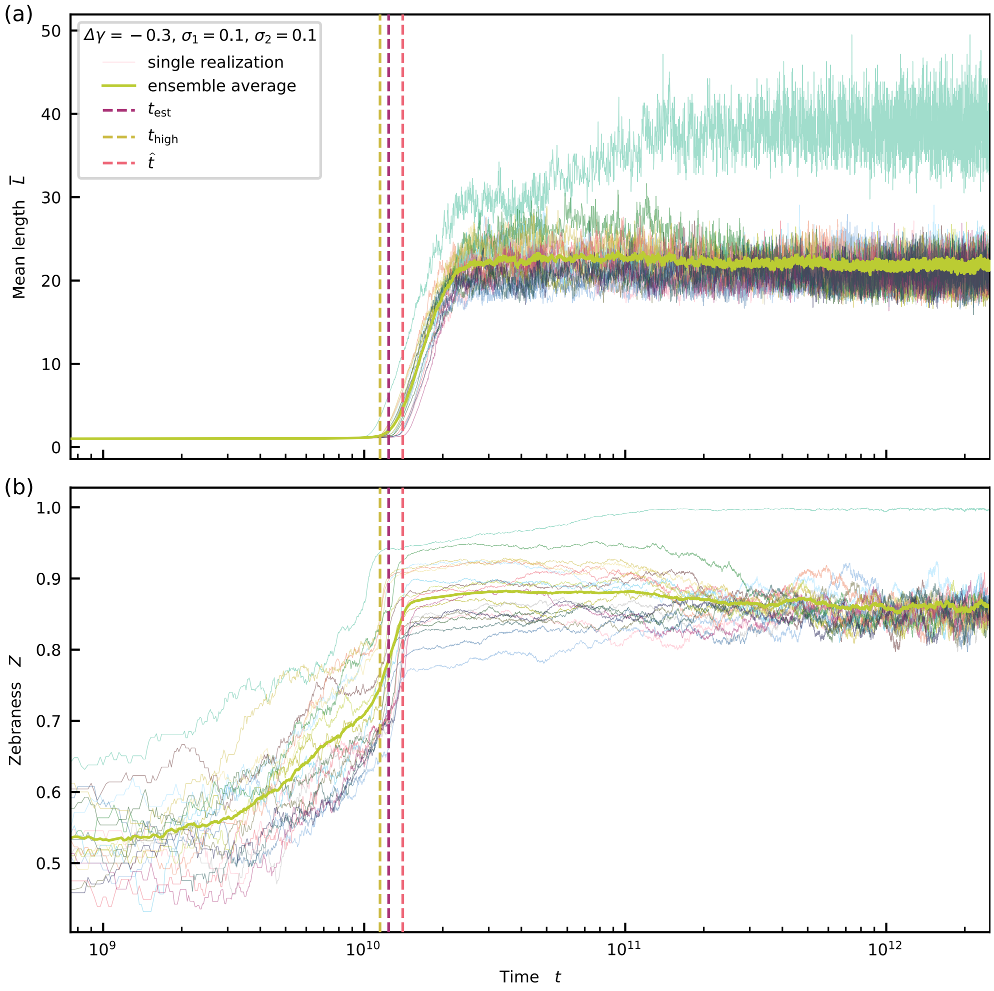

FIG. S23. Evolution of the mean length $\overline{L}$ and system-level zebraness Z for $\Delta\gamma = -0.3$ and $\sigma_1 = \sigma_1 = 0.1$ (weak kinetic stalling). While 19 out of 20 trajectories reach a non-pure zebra state, one trajectory converges to a pure zebra state. The relative deviations of t_{est} from t_{high} and $\widehat{t}$ are -7.8% and 11.7%. (See also Figure 5 in the main text.)

S7. ONSET OF GROWTH — DETAILS

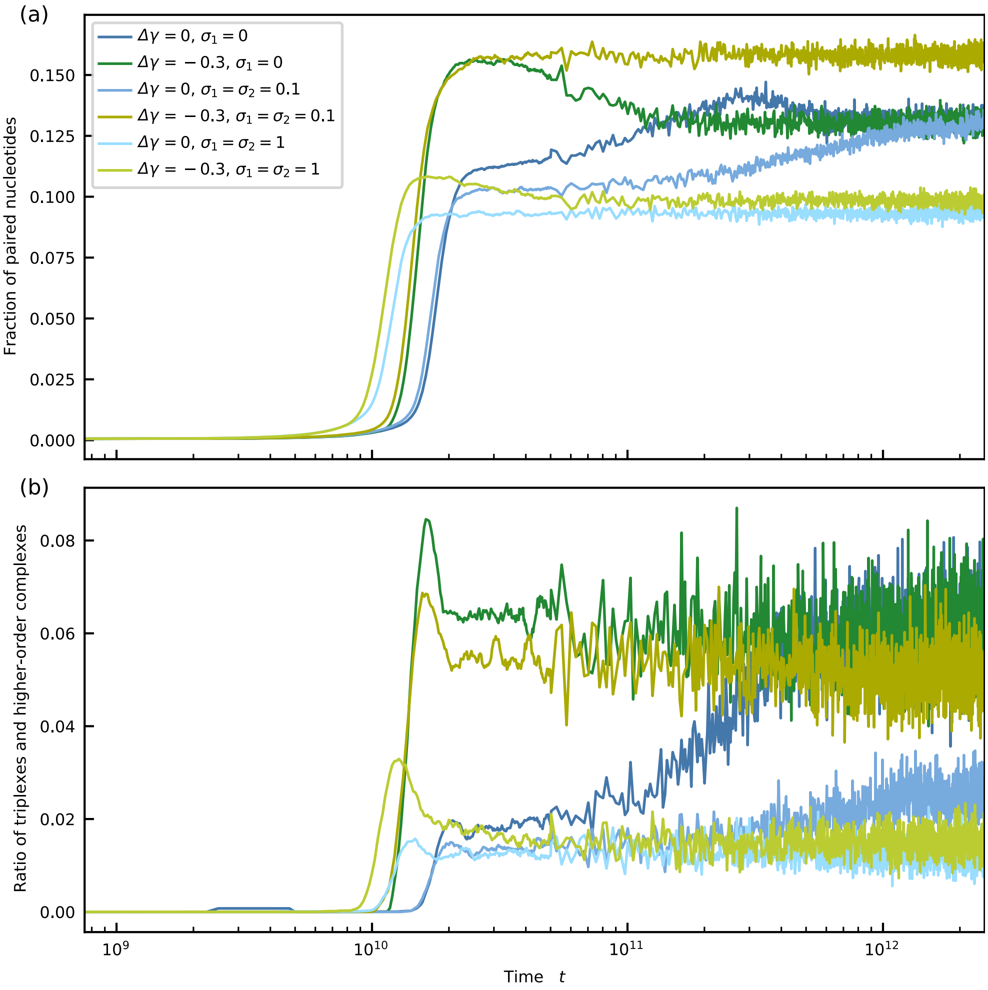

FIG. S24. (a) Evolution of the number of paired nucleotides for various values of $\Delta\gamma$ and σ_1 and $\sigma_1 = 0.1$ normalized with respect to the total number of nucleotides. Initially, the fraction of bound nucleotides is negligible. (b) Evolution of the ratio of triplexes and higher-order complexes, i.e., complexes composed of more than three strands. For early times, we can neglect higher-order complexes.
