# Supplementary figures and images for: Thermodynamic and Kinetic Sequence Selection in Enzyme-Free Polymer Self-Assembly inside a Non-equilibrium RNA Reactor
Source: Life (Basel). 2022 Apr 10;12(4):567. doi: 10.3390/life12040567 (PMC9032526; doi:10.3390/life12040567)

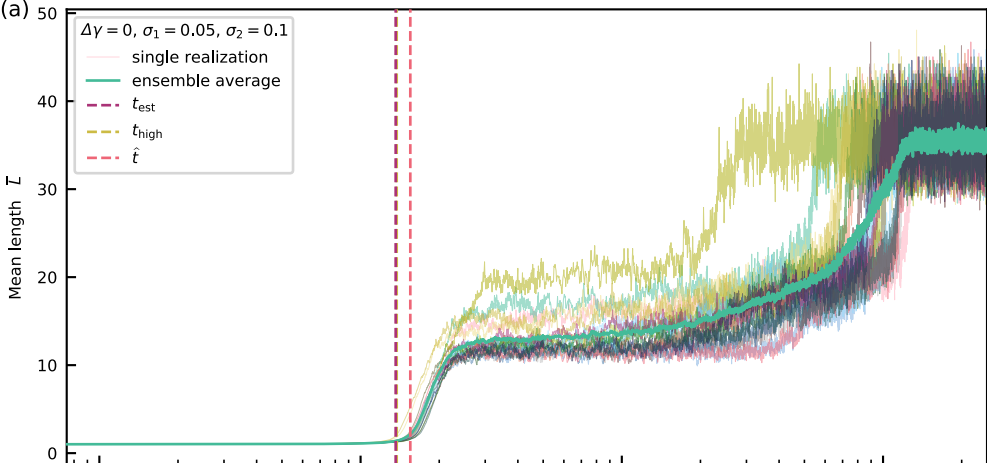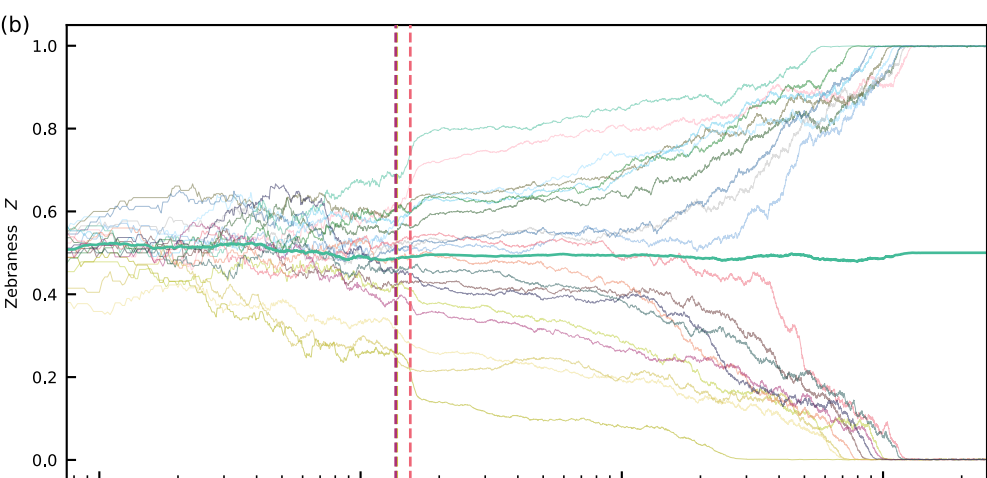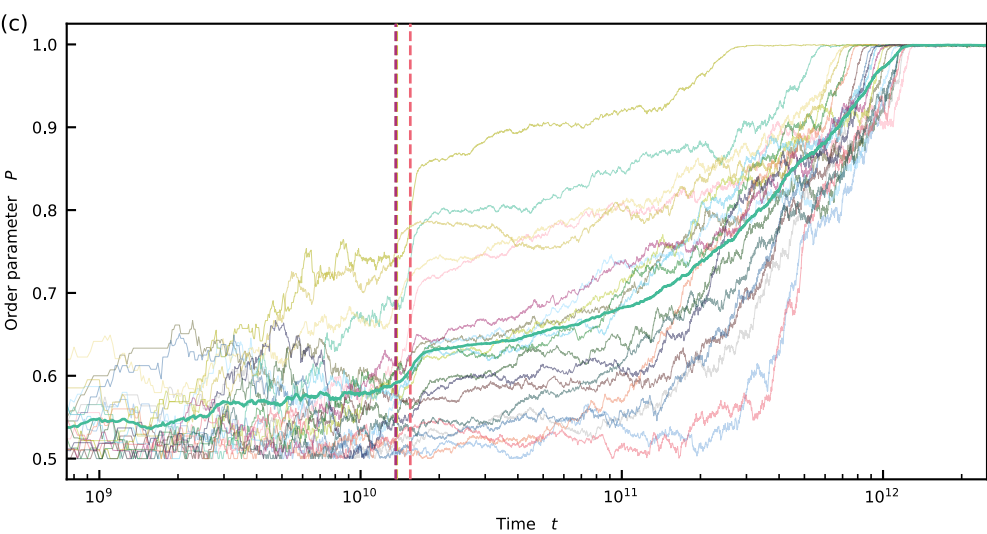

Supplement: Supplementary file 1 [file life-12-00567-s001.zip › FIGS10_LIFE.pdf]

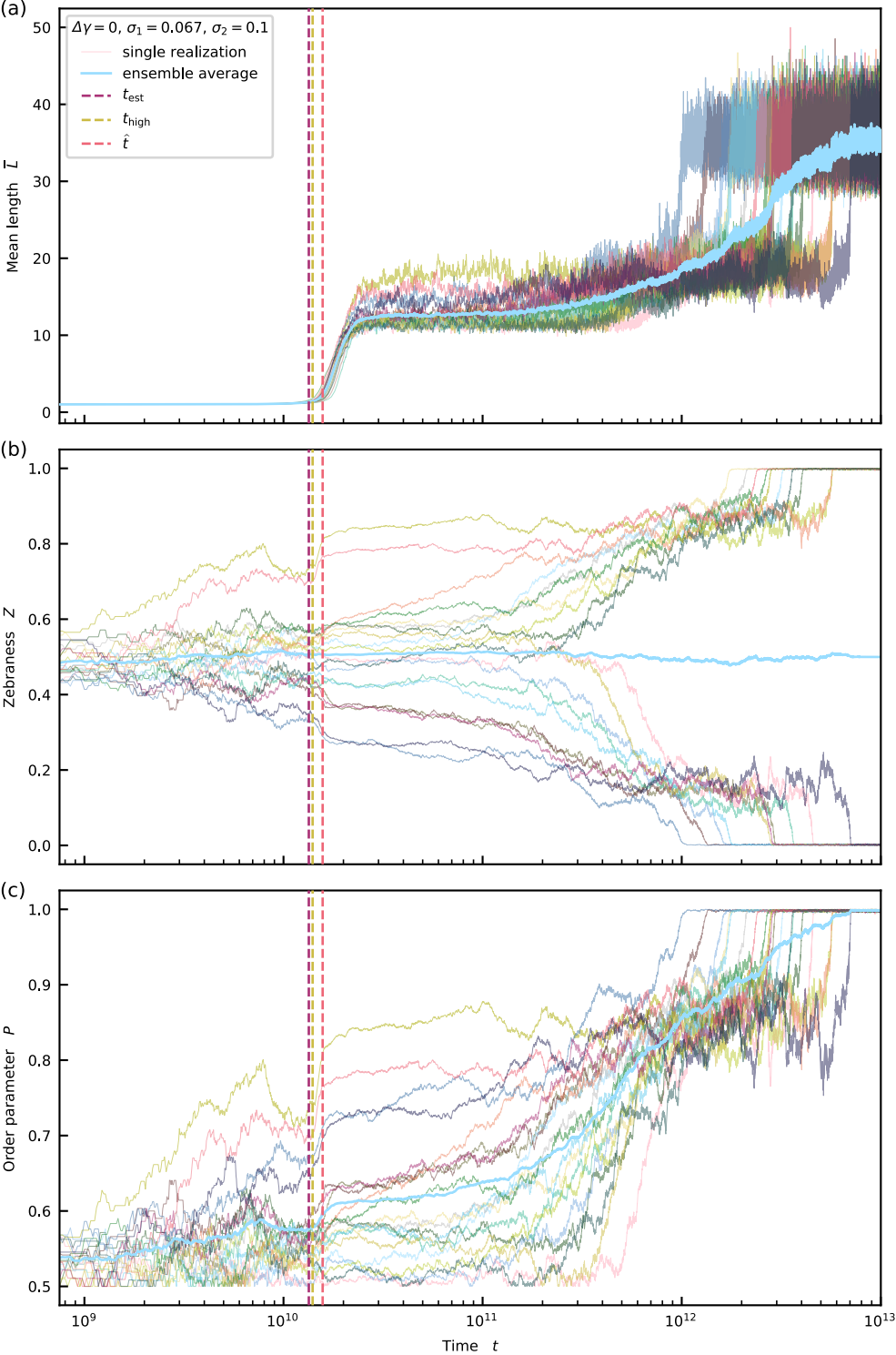

Supplement: Supplementary file 1 [file life-12-00567-s001.zip › FIGS11_LIFE.pdf]

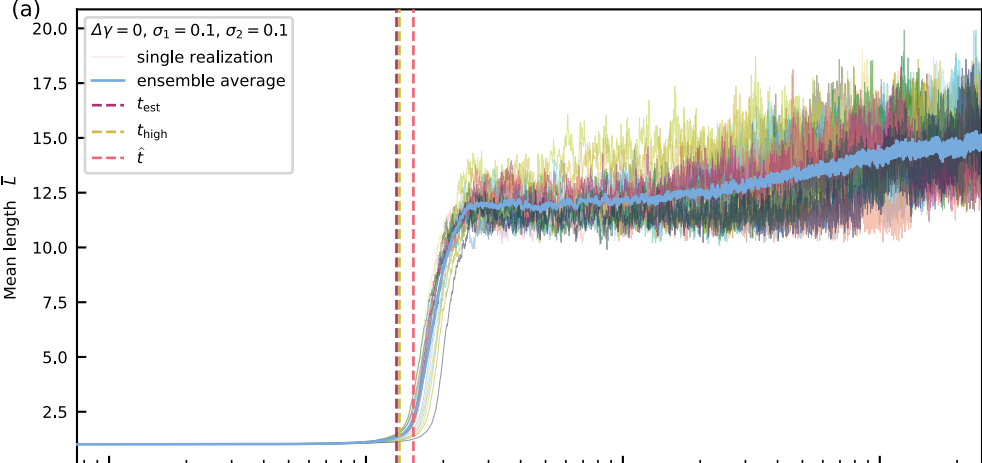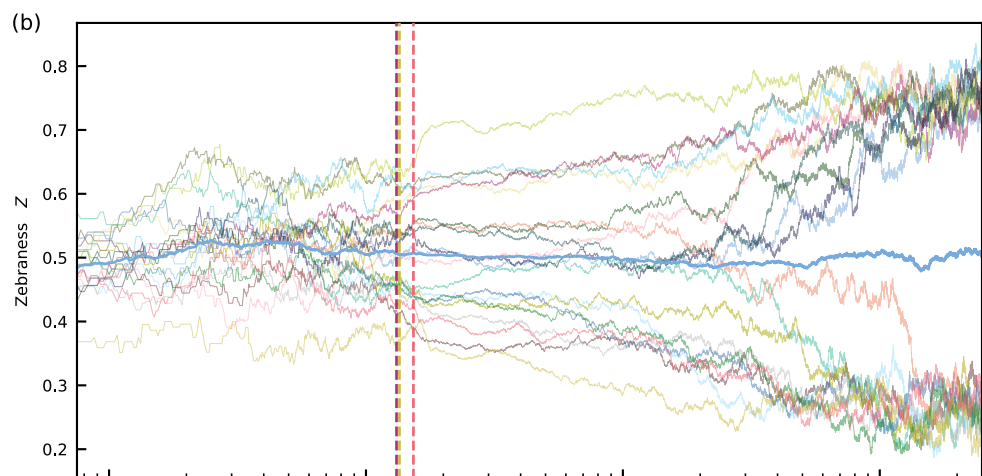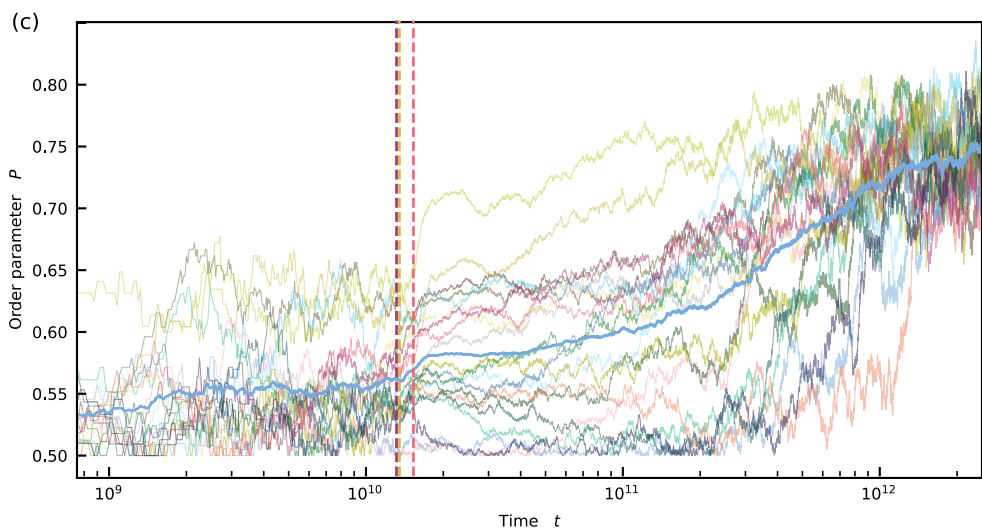

Supplement: Supplementary file 1 [file life-12-00567-s001.zip › FIGS12_LIFE.pdf]

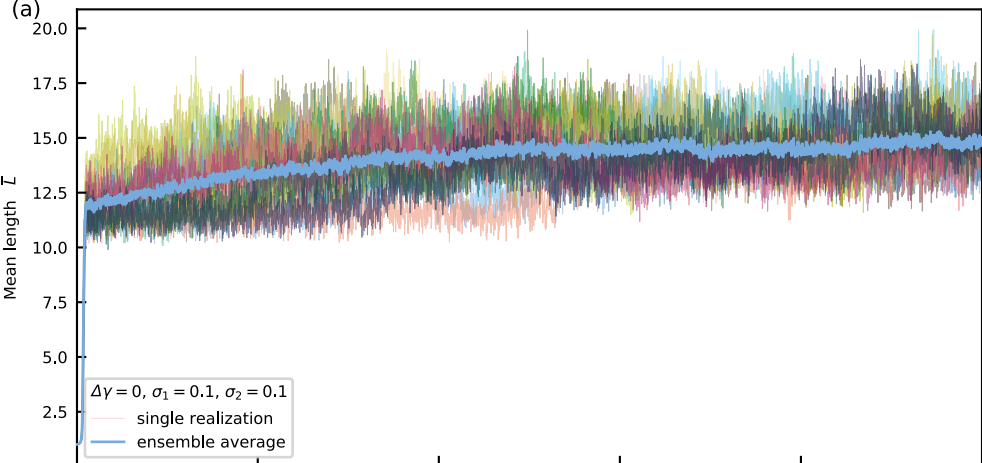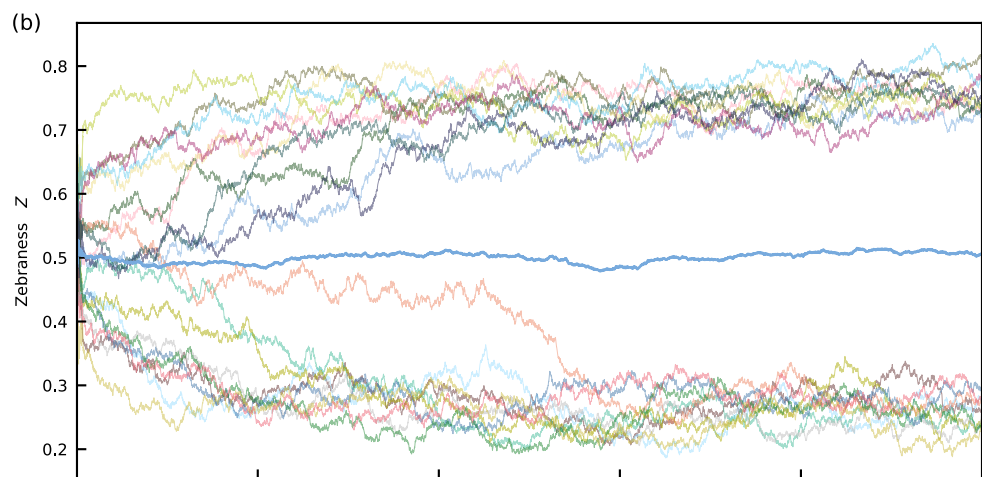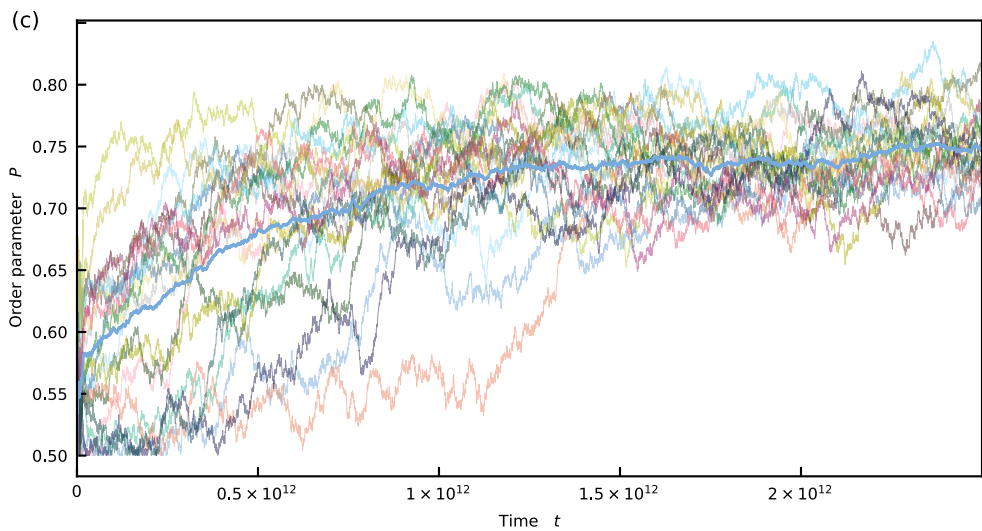

Supplement: Supplementary file 1 [file life-12-00567-s001.zip › FIGS13_LIFE.pdf]

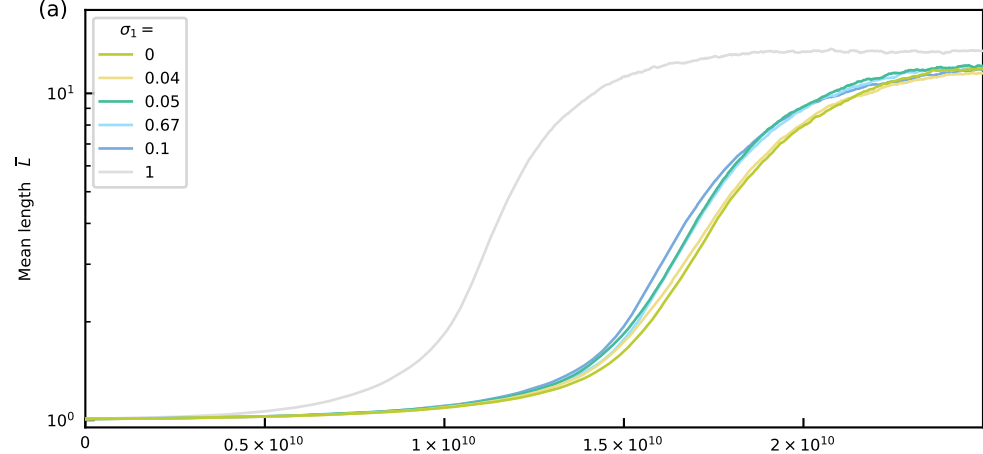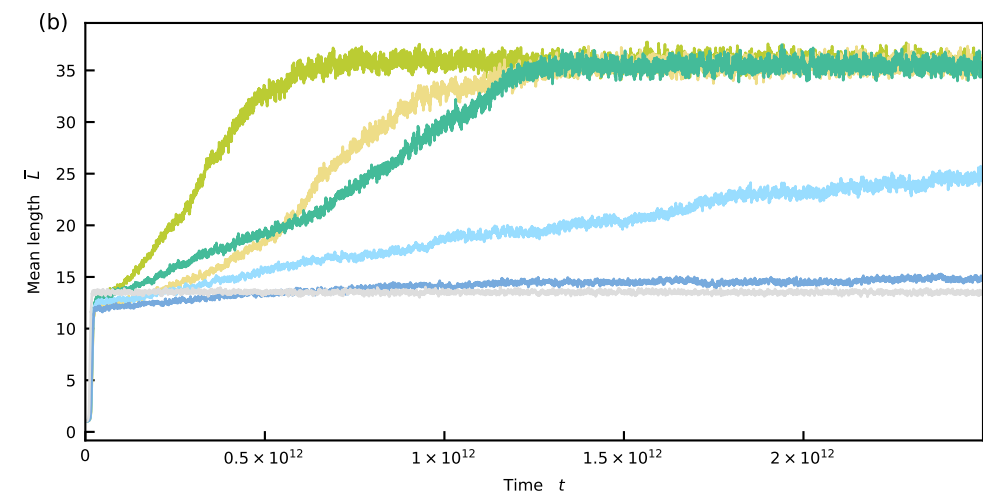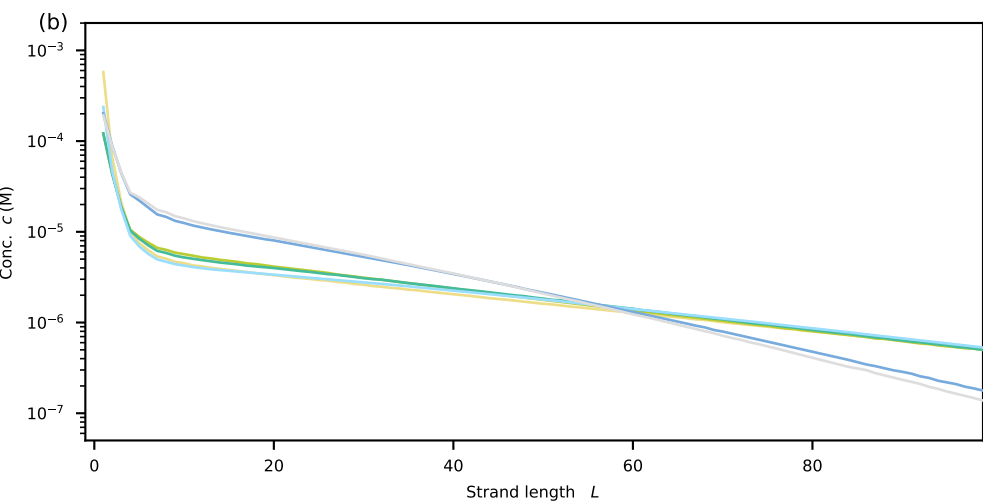

Supplement: Supplementary file 1 [file life-12-00567-s001.zip › FIGS14_LIFE.pdf]

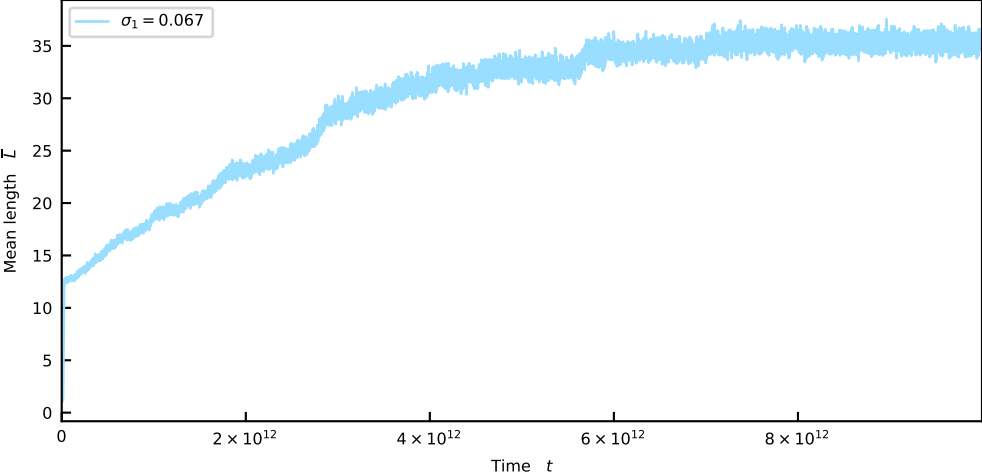

Supplement: Supplementary file 1 [file life-12-00567-s001.zip › FIGS15_LIFE.pdf]

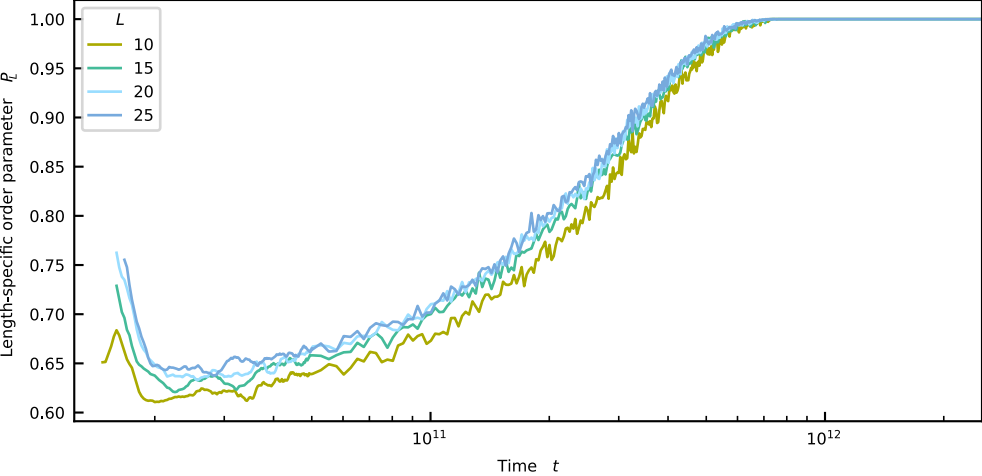

Supplement: Supplementary file 1 [file life-12-00567-s001.zip › FIGS17_LIFE.pdf]

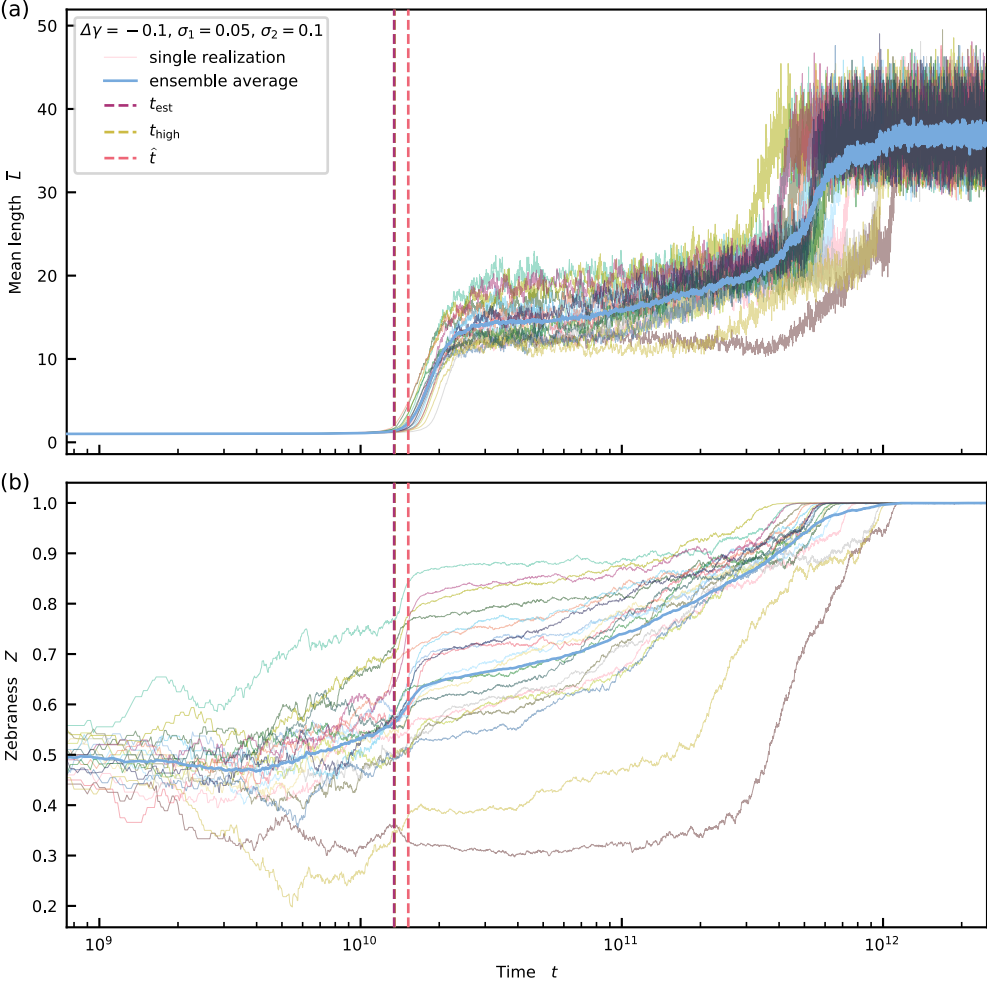

Supplement: Supplementary file 1 [file life-12-00567-s001.zip › FIGS18_LIFE.pdf]

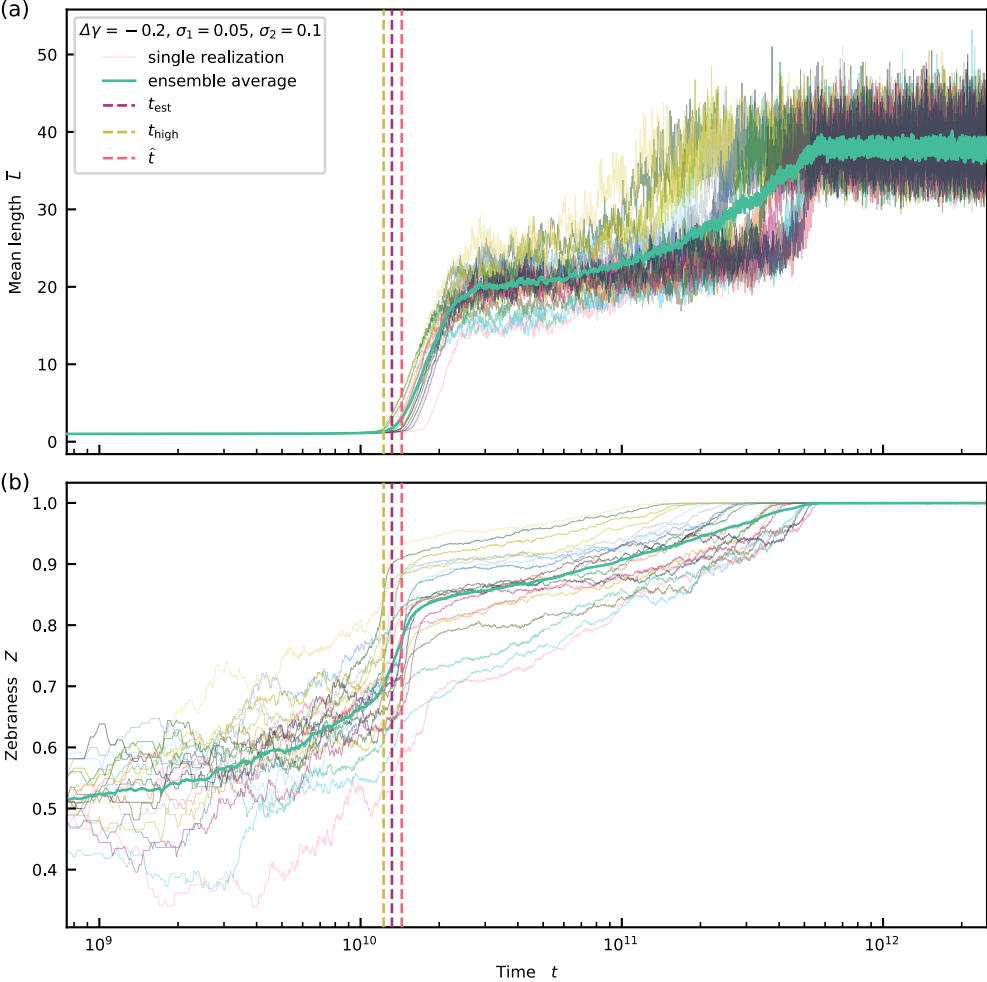

Supplement: Supplementary file 1 [file life-12-00567-s001.zip › FIGS19_LIFE.pdf]

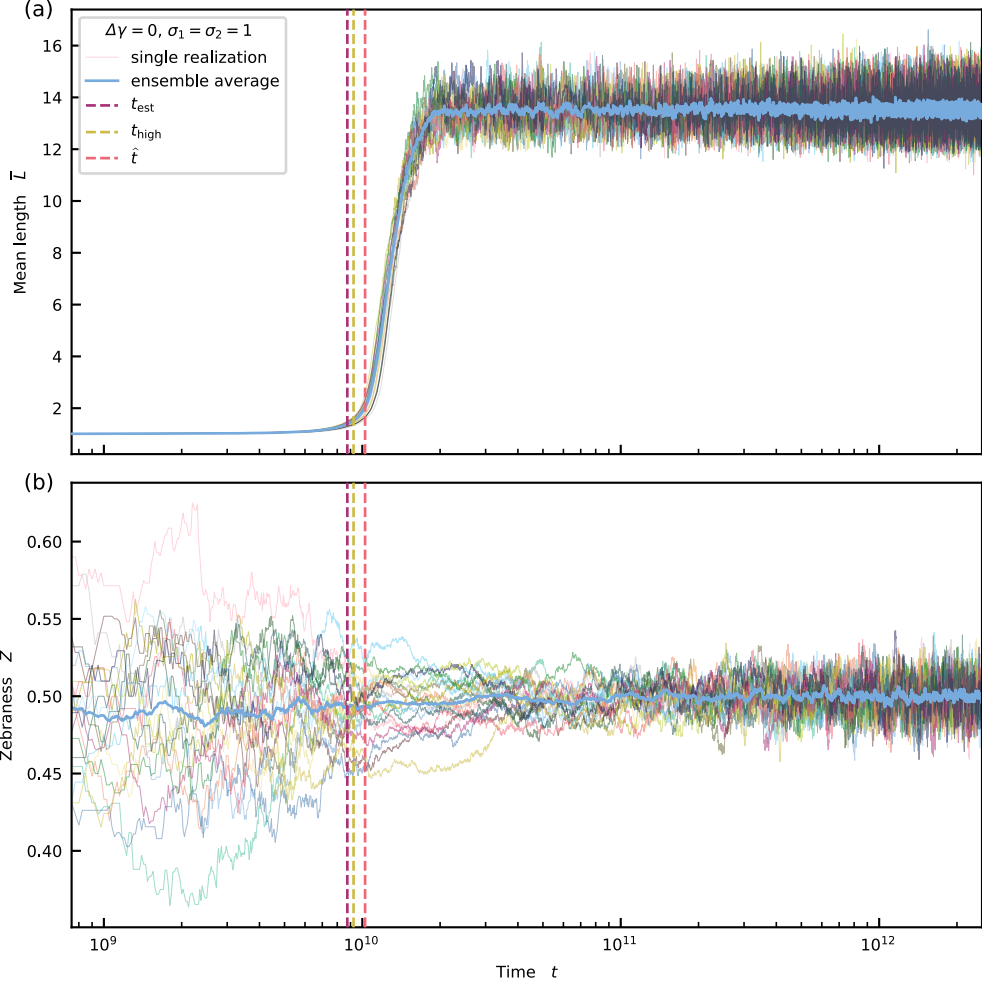

Supplement: Supplementary file 1 [file life-12-00567-s001.zip › FIGS1_LIFE.pdf]

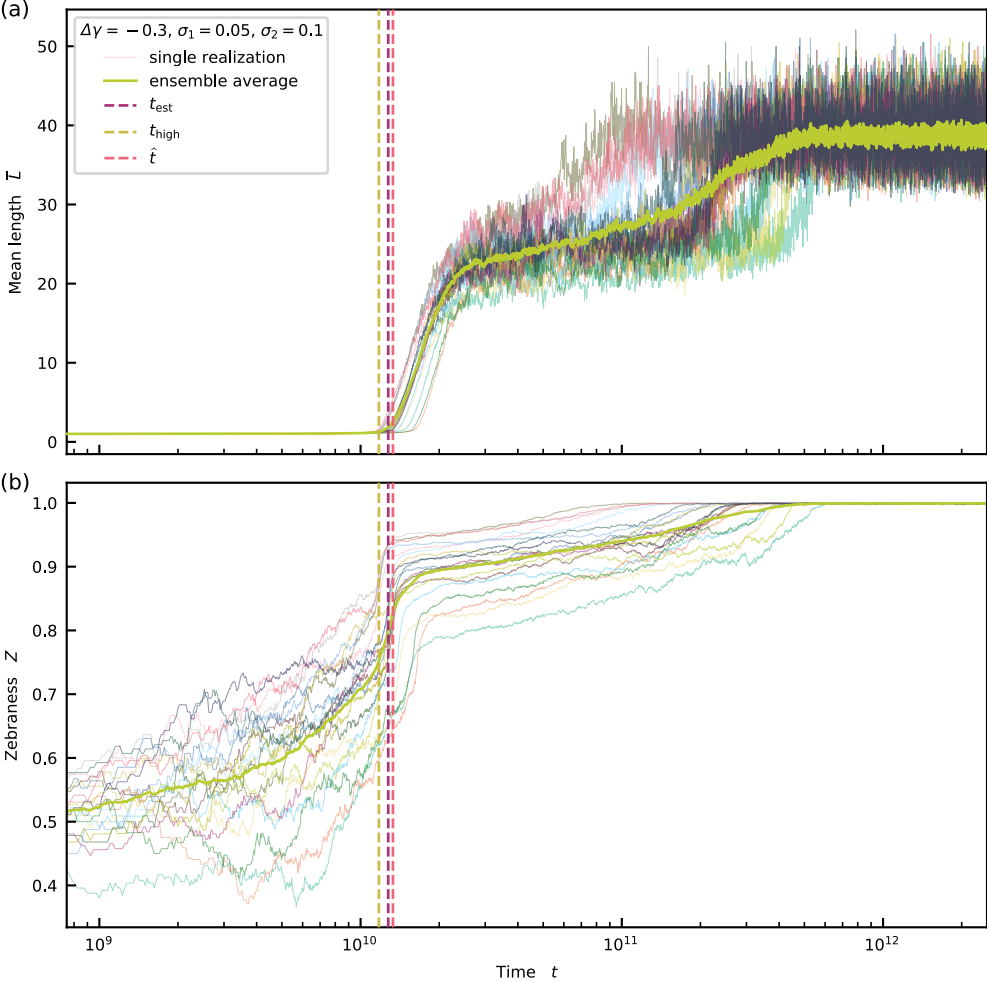

Supplement: Supplementary file 1 [file life-12-00567-s001.zip › FIGS20_LIFE.pdf]

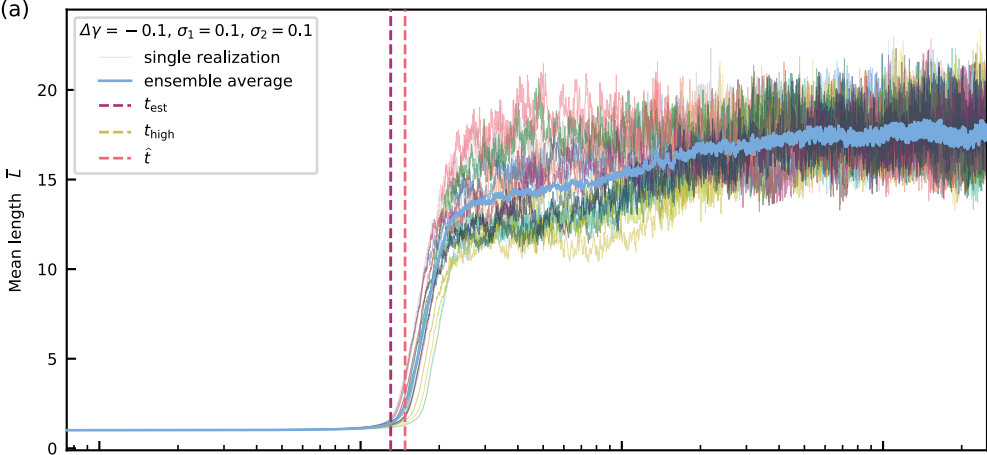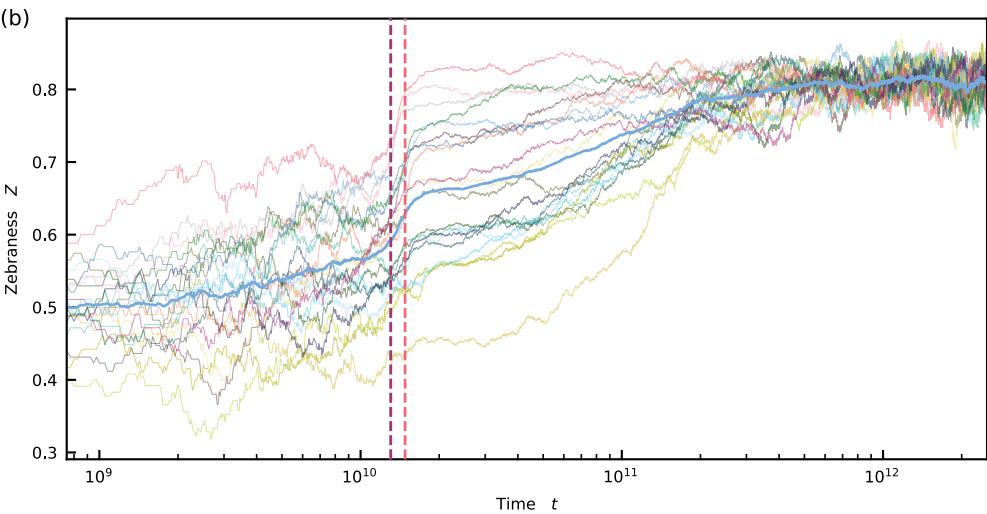

Supplement: Supplementary file 1 [file life-12-00567-s001.zip › FIGS21_LIFE.pdf]

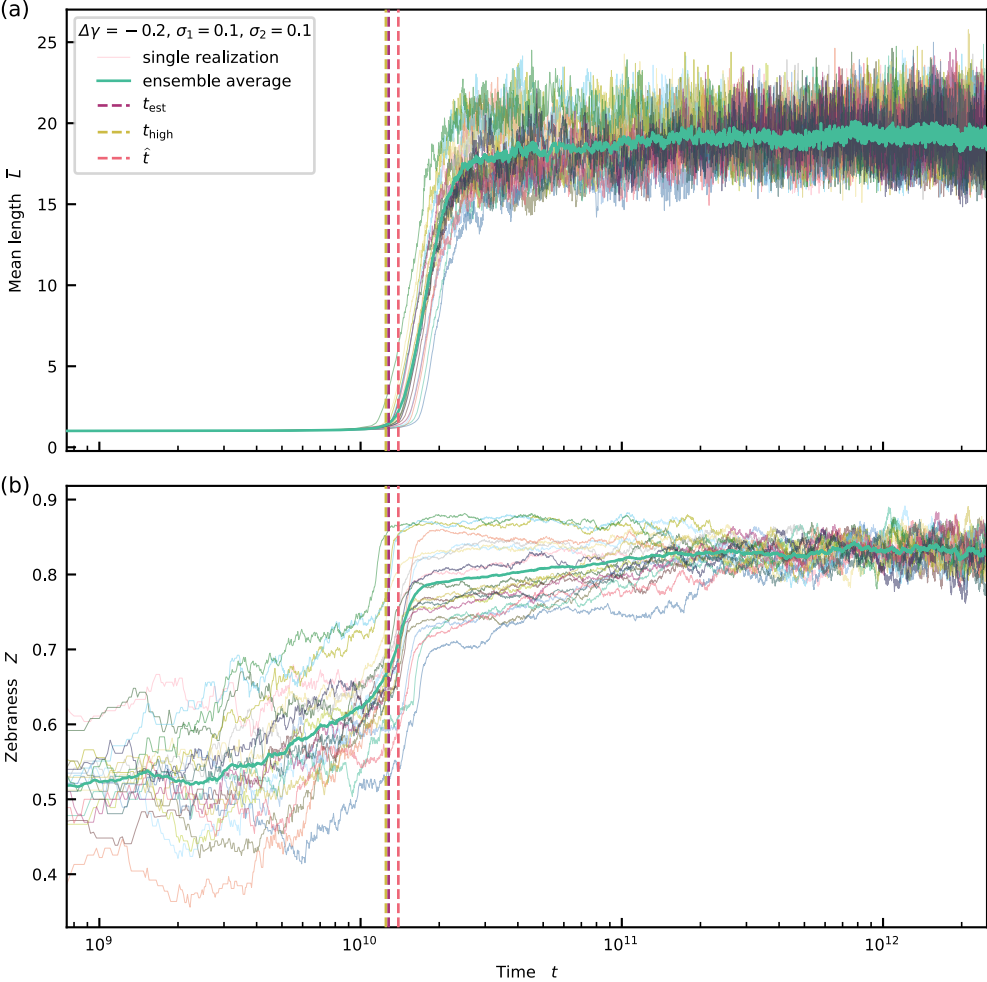

Supplement: Supplementary file 1 [file life-12-00567-s001.zip › FIGS22_LIFE.pdf]

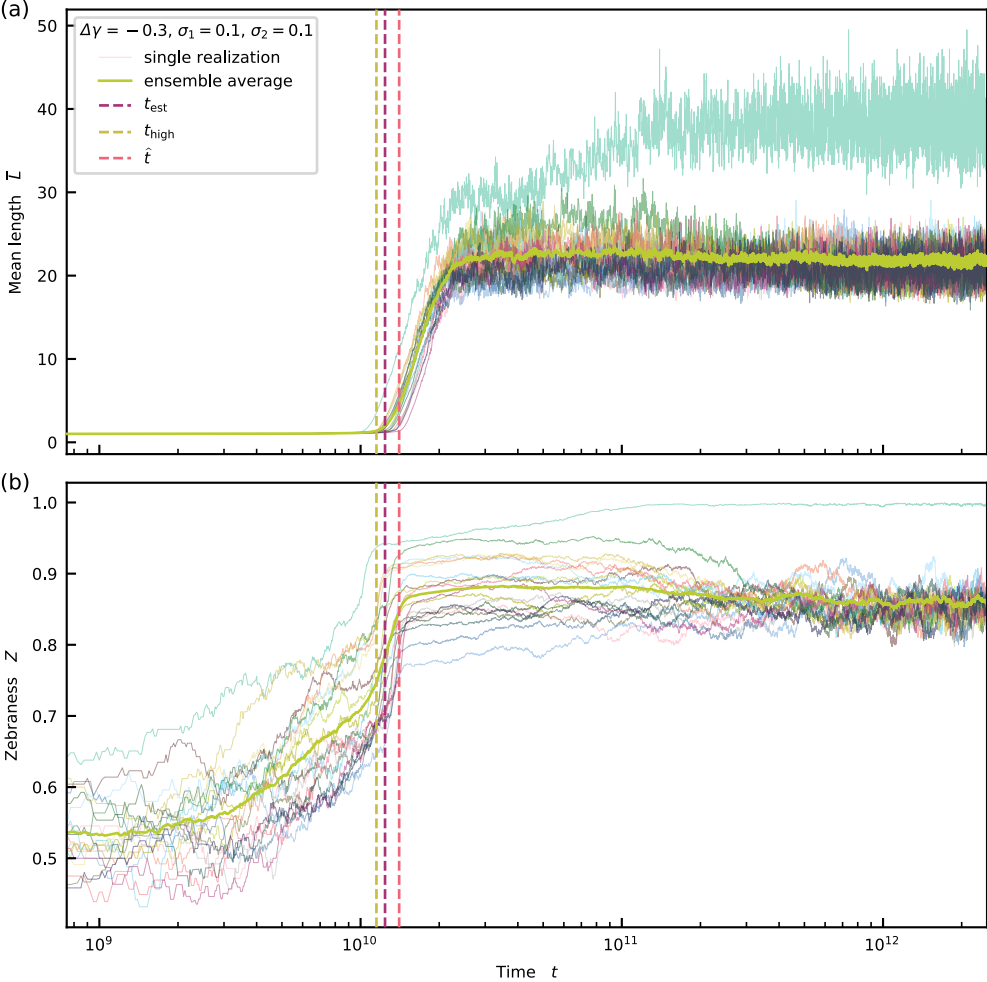

Supplement: Supplementary file 1 [file life-12-00567-s001.zip › FIGS23_LIFE.pdf]

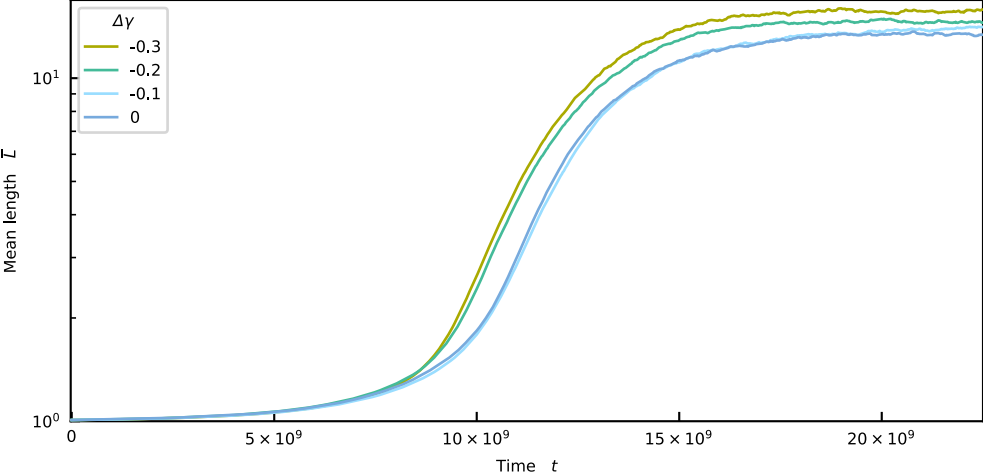

Supplement: Supplementary file 1 [file life-12-00567-s001.zip › FIGS2_LIFE.pdf]

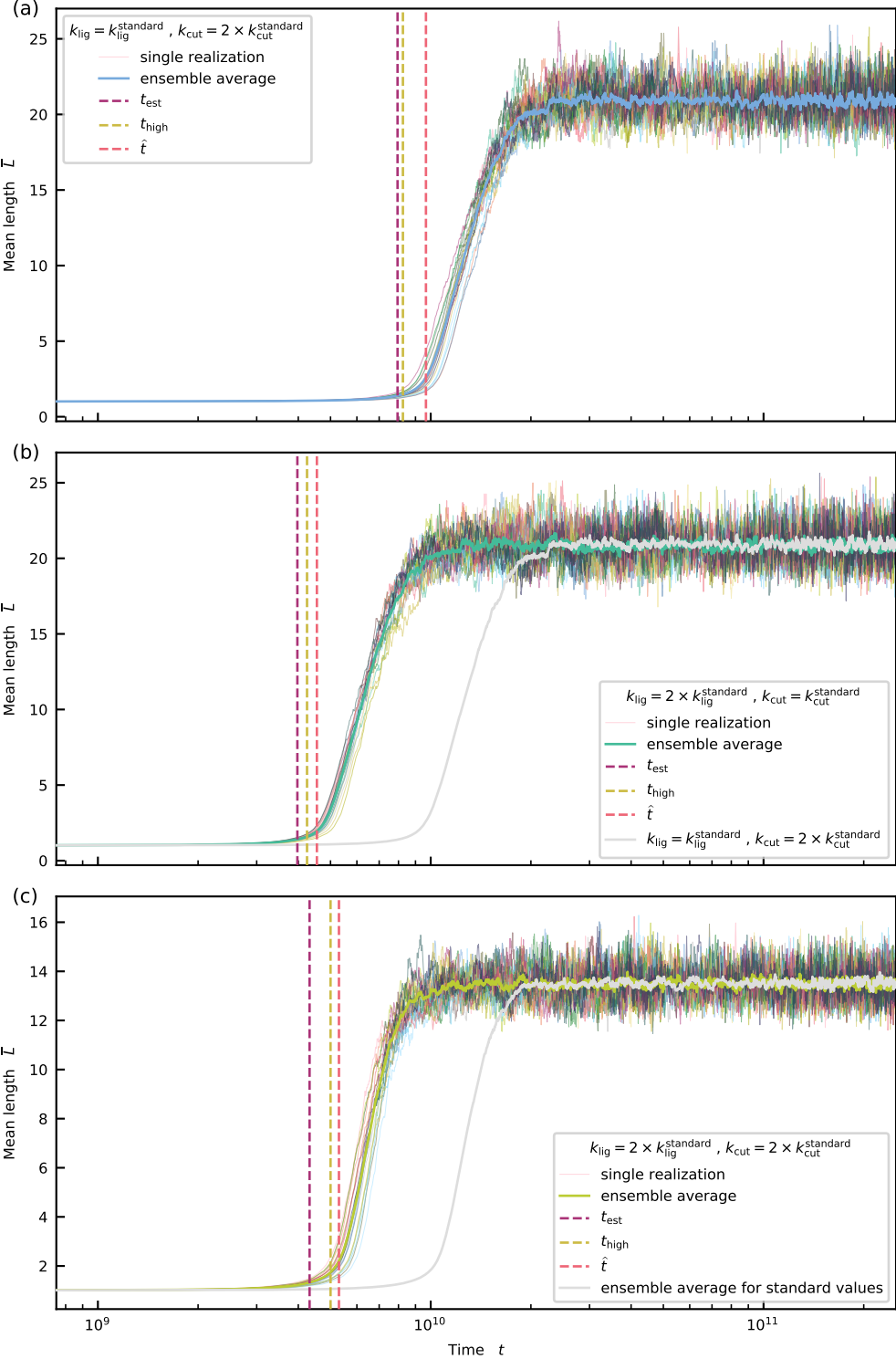

Supplement: Supplementary file 1 [file life-12-00567-s001.zip › FIGS3_LIFE.pdf]

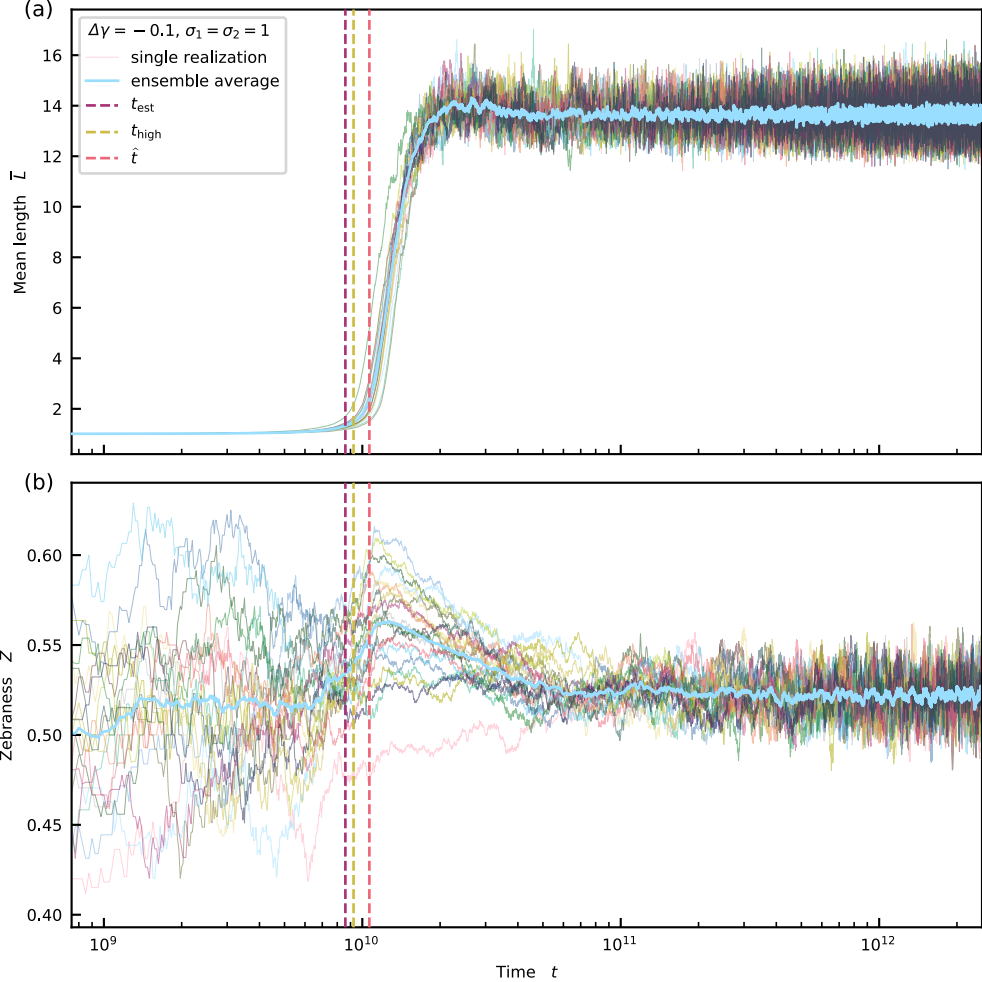

Supplement: Supplementary file 1 [file life-12-00567-s001.zip › FIGS4_LIFE.pdf]

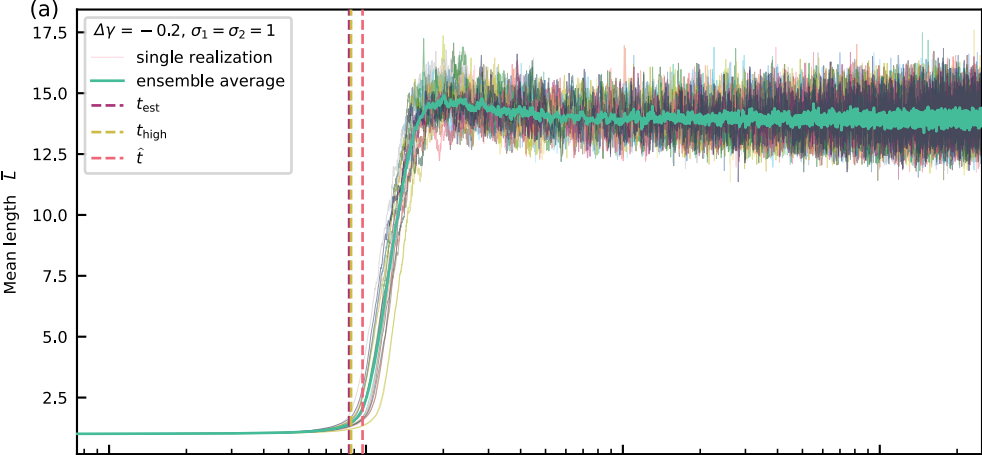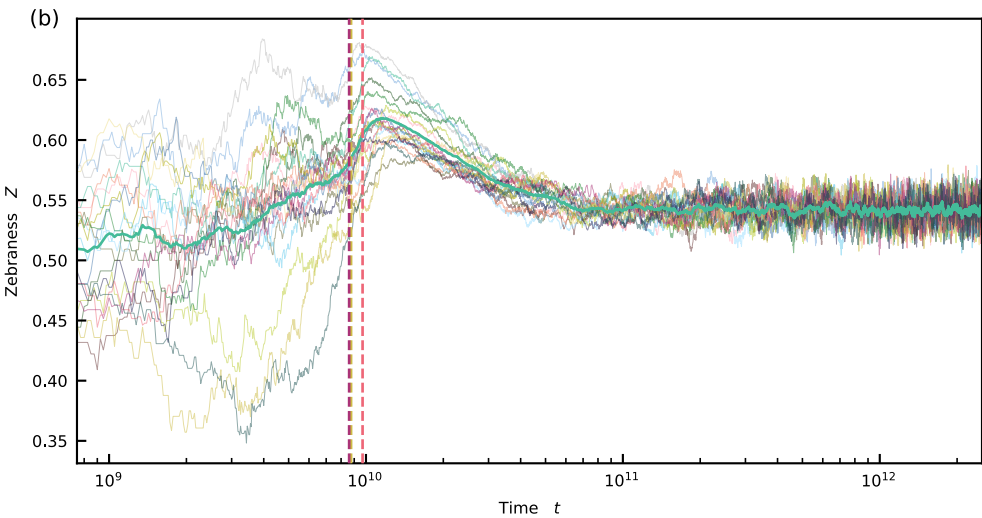

Supplement: Supplementary file 1 [file life-12-00567-s001.zip › FIGS5_LIFE.pdf]

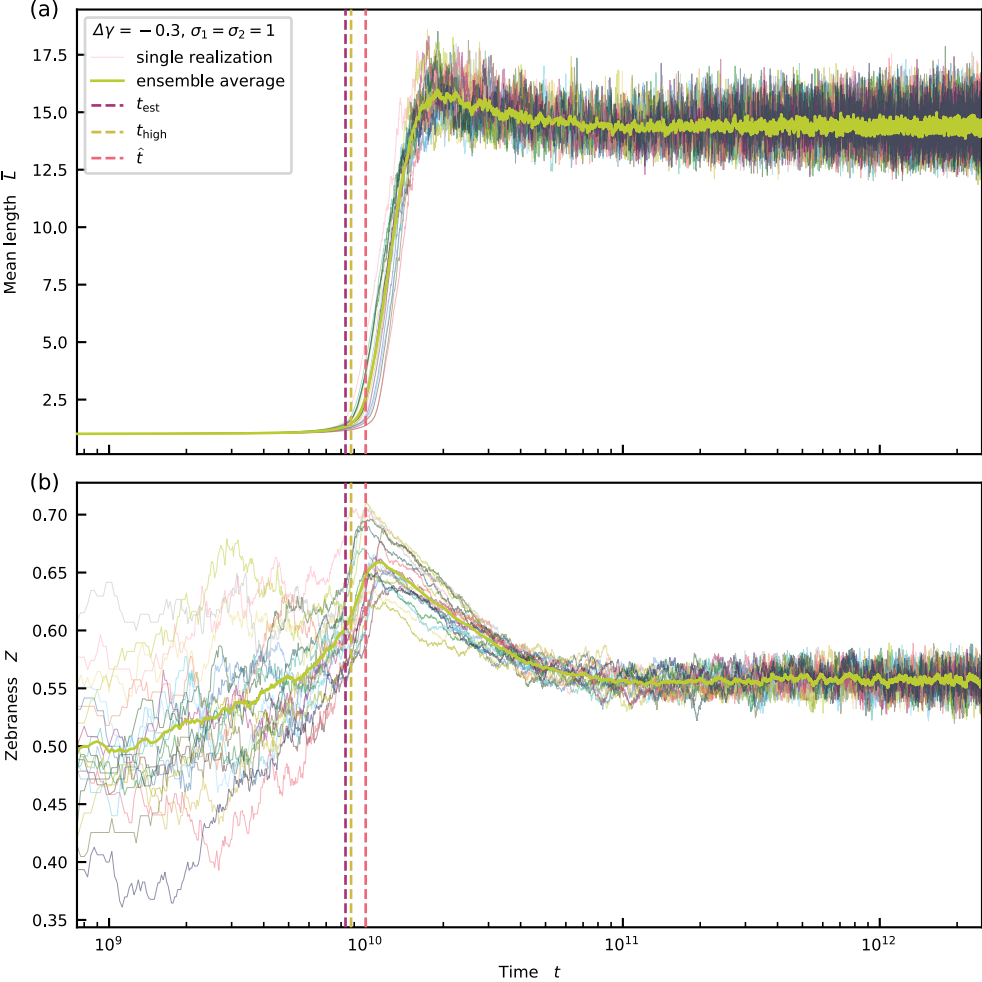

Supplement: Supplementary file 1 [file life-12-00567-s001.zip › FIGS6_LIFE.pdf]

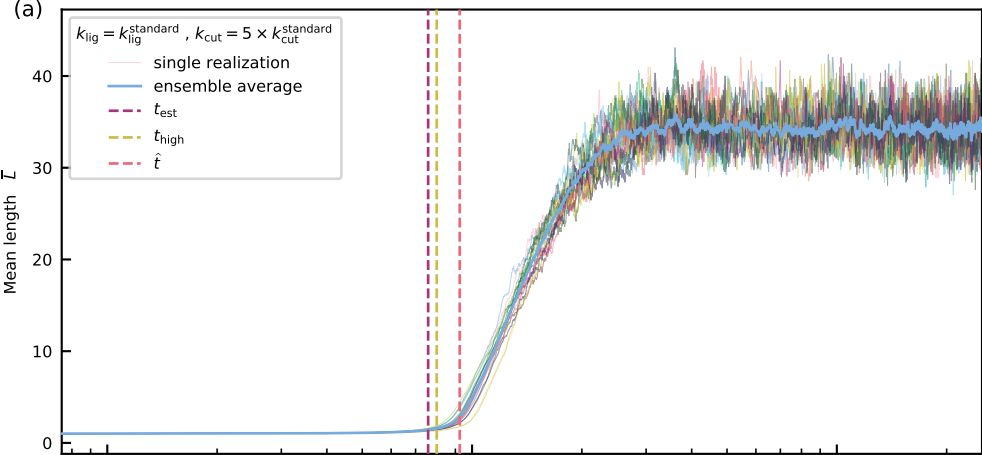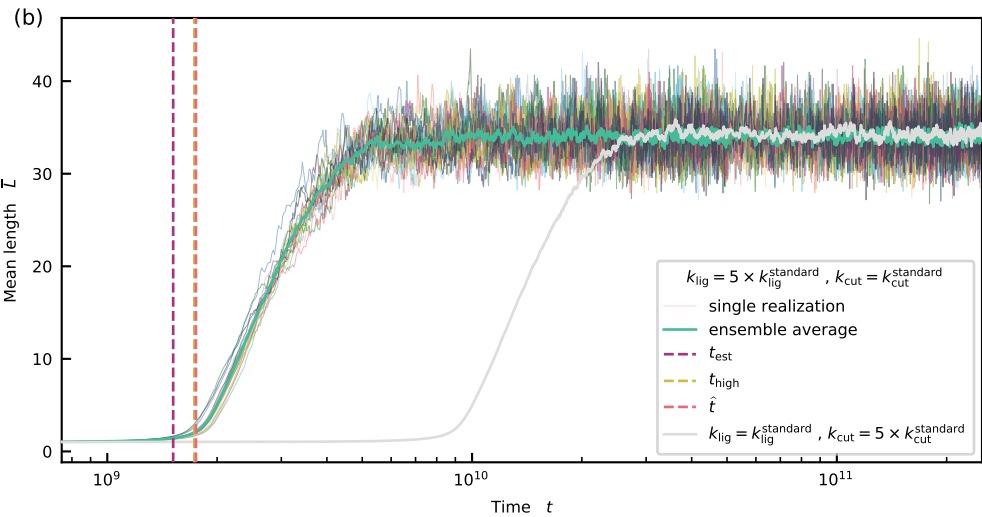

Supplement: Supplementary file 1 [file life-12-00567-s001.zip › FIGS7_LIFE.pdf]

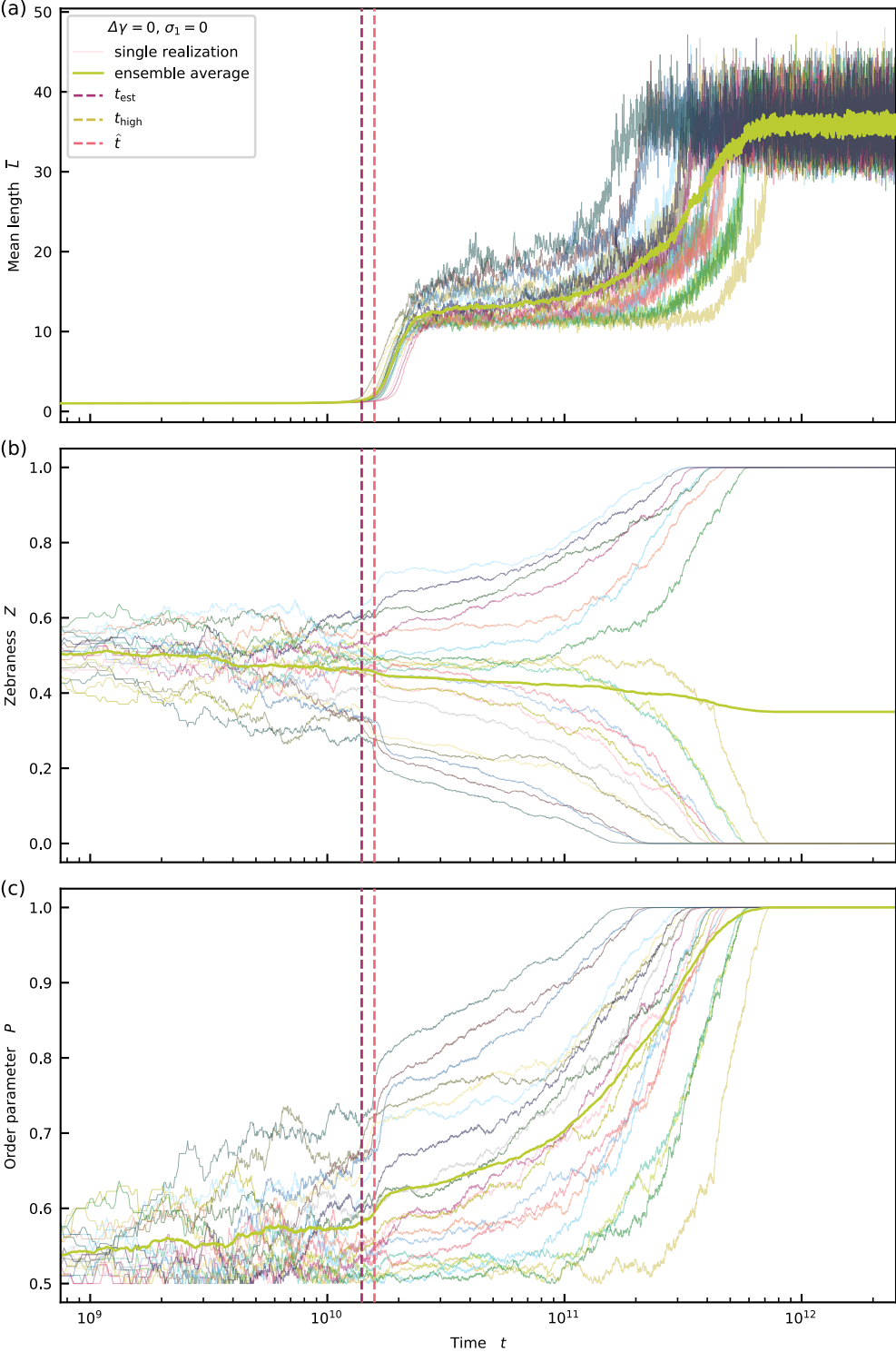

Supplement: Supplementary file 1 [file life-12-00567-s001.zip › FIGS8_LIFE.pdf]

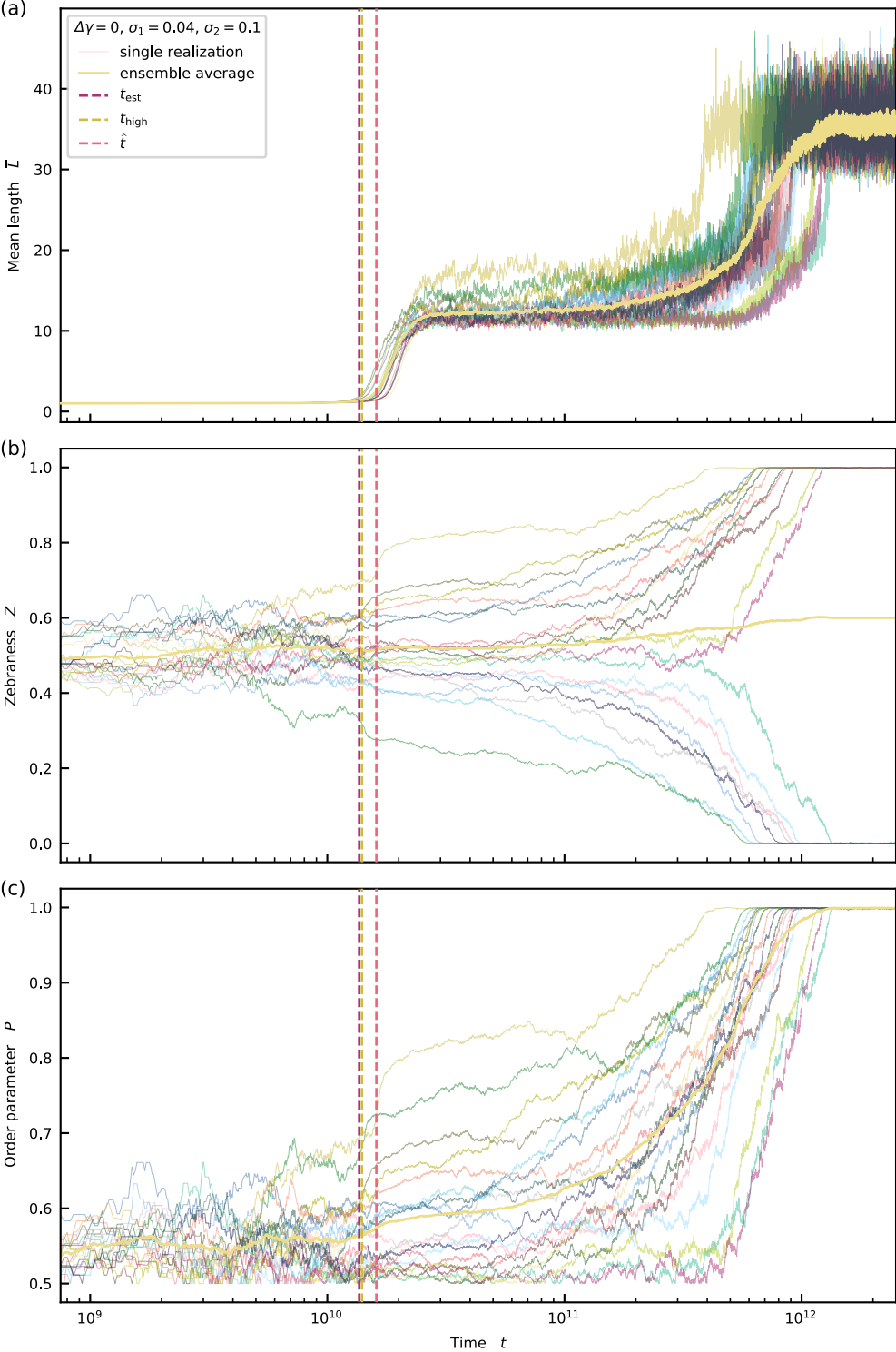

Supplement: Supplementary file 1 [file life-12-00567-s001.zip › FIGS9_LIFE.pdf]

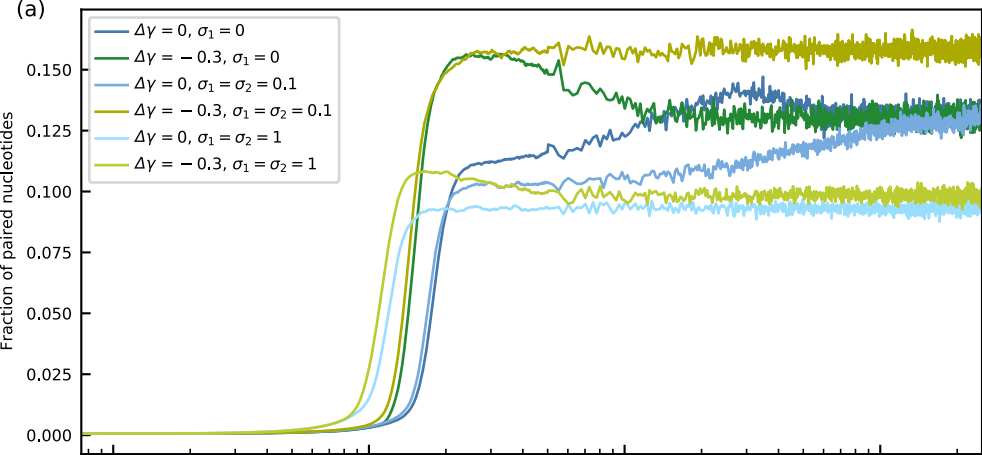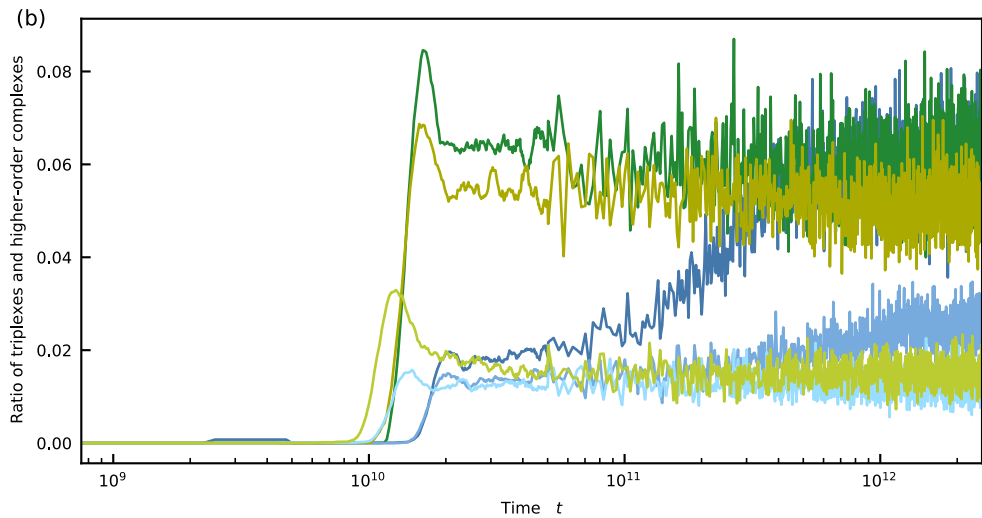

Supplement: Supplementary file 1 [file life-12-00567-s001.zip › FIGSX1_LIFE.pdf]

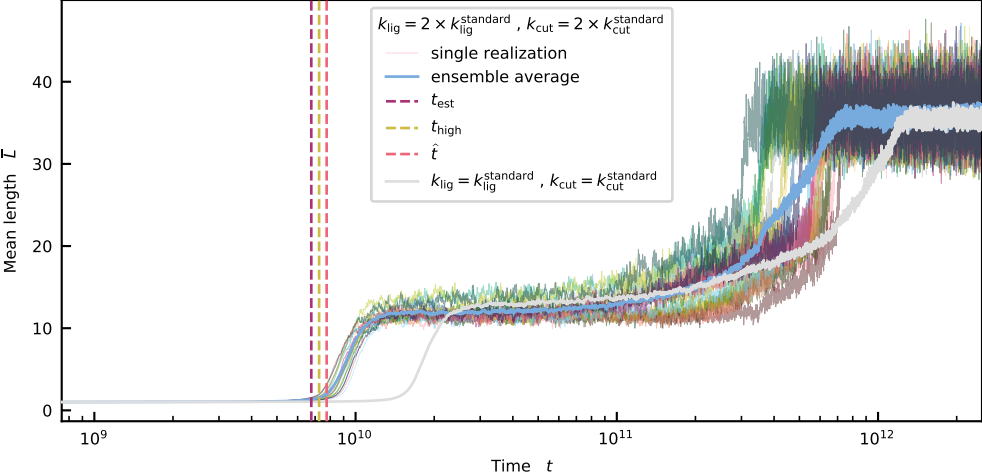

Supplement: Supplementary file 1 [file life-12-00567-s001.zip › FIGSY1_LIFE.pdf]
